# Clinical features, disease severity, and outcomes of pertussis in hospitalized children: A retrospective study from Serbia

**DOI:** 10.1371/journal.pone.0352498

**Published:** 2026-06-26

**Authors:** Mihail Basa, Jelena Visekruna, Tijana Grba, Aleksandra Paripovic, Marija Marsenic, Vladimir Petrovic, Aleksandar Sovtic

**Affiliations:** 1 Department of Pulmonology, Mother and Child Health Care Institute of Serbia ‘Dr Vukan Cupic’, Belgrade, Serbia; 2 Department of Nephrology, Mother and Child Health Care Institute of Serbia ‘Dr Vukan Cupic’, Belgrade, Serbia; 3 Medical Faculty, University of Belgrade, Belgrade, Serbia; 4 General Hospital Berane, Berane, Montenegro; 5 Institute for Public Health of Vojvodina, Novi Sad, Serbia; 6 Faculty of Medicine, University of Novi Sad, Novi Sad, Serbia; Universidad Nacional de la Plata, ARGENTINA

## Abstract

**Background:**

Severe pertussis remains a major cause of morbidity and mortality in early infancy, particularly among unvaccinated and preterm infants who lack sufficient maternally derived antibody protection. This study aimed to identify clinical and laboratory predictors of severe disease and fatal outcomes in hospitalized infants and young children with PCR- or serologically confirmed pertussis.

**Methods:**

We conducted a retrospective study of 107 pediatric patients hospitalized with pertussis at a tertiary care center in Serbia between 2015 and 2025. Clinical characteristics, laboratory parameters, and outcomes were analyzed. Logistic regression—including Firth’s penalized models where appropriate—was used to explore predictors of PICU admission, mechanical ventilation (MV), and mortality. Viral coinfections were assessed using multiplex PCR, and leukocyte dynamics were monitored through standardized follow-up testing.

**Results:**

Thirteen children (12.1%) required PICU admission and 12 (11.2%) underwent MV. Five patients (4.7%) died; all were unvaccinated infants younger than 2 months of age. Prematurity was a strong independent predictor of PICU admission and MV. Higher absolute lymphocyte counts and an elevated neutrophil-to-lymphocyte ratio at admission were also associated with severe disease.

Viral coinfection was documented in 22.4% of patients and was associated with increased mortality. In exploratory analyses, both viral coinfection and elevated N/L ratio were associated with fatal outcome; however, these findings should be interpreted with caution due to the small number of events.

**Conclusions:**

Severe and fatal pertussis predominantly affected infants in the first months of life, especially those born preterm and those without maternal or postnatal vaccine-derived protection. Viral coinfection was associated with worse clinical outcomes. These findings highlight the importance of early identification of high-risk patients and support preventive strategies targeting early infancy, including maternal pertussis immunization.

## Introduction

Pertussis is a highly contagious respiratory disease characterized by a prolonged paroxysmal cough and the potential for life-threatening complications, including respiratory failure, pulmonary hypertension, and intracranial hypertension [1]. Although it can affect individuals across all age groups, the risk of severe outcomes is particularly pronounced in infants and young children [1]. Widespread implementation of routine immunization programs has substantially reduced the overall burden of disease [2]. However, pertussis remains a clinical concern due to its continued occurrence among unvaccinated or incompletely vaccinated individuals. Reports of localized outbreaks—especially among neonates and adolescents—have prompted renewed attention to the adequacy of current immunization strategies and the effectiveness of epidemiologic surveillance systems [3]. In recent years, a resurgence of pertussis has been observed globally, particularly in the post-COVID-19 period, with outbreaks reported across Europe and North America. This re-emergence has been linked to multiple factors, including disruptions in routine immunization, declining vaccination coverage, waning vaccine-induced immunity, and insufficient uptake of booster doses [4]. These observations highlight the need to reevaluate both the timing and structure of vaccination schedules, as well as protocols for monitoring affected patients and their close contacts.

Despite the success of routine childhood immunization programs, several gaps in pertussis prevention remain. In many countries, national vaccination schedules do not include maternal immunization during pregnancy, thereby limiting the transplacental transfer of protective antibodies and leaving newborns vulnerable until the administration of the first pertussis-containing vaccine dose [2,3]. In the Republic of Serbia, pertussis vaccination during pregnancy is recommended by national expert bodies; however, it is not included in the mandatory national immunization program and is therefore not systematically implemented.

Therapeutic options for pertussis remain limited. While macrolide antibiotics have demonstrated clinical benefit during the catarrhal and early paroxysmal phases by reducing bacterial load and transmission, the broad spectrum of symptomatic interventions employed in later stages has shown minimal impact on disease progression or outcome [5,6]. Consequently, increasing attention is being directed toward the early identification of patients at risk for severe disease—particularly those requiring hospitalization and, in many cases, admission to pediatric intensive care units (PICUs) [7]. Within the PICU setting, treatment strategies are primarily focused on managing the critical components of severe pertussis, including the provision of mechanical ventilation and targeted interventions to reduce extreme lymphocytosis, often accompanied by adjunctive anti-inflammatory measures aimed at mitigating the systemic inflammatory response [8].

While marked lymphocytosis and neonatal age have been shown to be key predictors of mortality in the pediatric intensive care unit (PICU), limited data exist regarding early warning signs of deterioration in children who were initially stable and admitted to general pulmonary wards [8]. Particular attention is given to indicators that precede transfer to PICU. Early recognition of such parameters is essential to enable timely escalation to intensive therapeutic modalities [9–11].

The objective of this study is to present the clinical experience and treatment outcomes of a national pediatric referral center in managing hospitalized children diagnosed with pertussis, as well as to identify predictors of clinical deterioration that necessitate PICU admission, with a focused analysis of factors associated with severe disease courses — such as the requirement for mechanical ventilation, exchange transfusion and renal replacement therapy.

## Methods

This retrospective study analyzed medical records of pediatric patients diagnosed with pertussis who were treated in inpatient settings at the Mother and Child Health Care Institute of Serbia, Belgrade, Serbia between March 2015 and March 2025. All eligible patients hospitalized during the study period were consecutively included. Patients were identified through hospital medical records based on confirmed pertussis diagnosis. The diagnosis of pertussis was established based on characteristic clinical presentation supported by laboratory findings and confirmed by polymerase chain reaction (PCR) testing, or by serology in patients evaluated more than 21 days after symptom onset [12]. Although hospitalization decisions were made on an individual basis, the primary goal of outpatient evaluation — conducted both at the Institute’s Emergency and Admission Department and, in some cases, at secondary healthcare institutions referring patients to our center — was to identify children at high risk for severe clinical progression and complications. These included: preterm infants; children within the first three months of life (prior to initiation of vaccination or with incomplete immunization); patients presenting with pronounced symptoms such as whooping cough and apneic episodes; those with marked leukocytosis and lymphocytosis (≥10,000 lymphocytes/μL of blood); and children exhibiting clear signs of respiratory distress and poor general condition. Prematurity was defined as birth before 37 completed weeks of gestation. Vaccination status was categorized as follows: children were considered unvaccinated if they had not received any pertussis-containing vaccine; partially vaccinated if they had received at least one but not all age-appropriate doses; and fully vaccinated if they had received all doses appropriate for their age according to the national immunization schedule.

The study was conducted in accordance with the Declaration of Helsinki and approved by the Institutional Ethics Committee of Mother and Child Health Care Institute of Serbia (protocol code 8/131; date of approval: 02/11/2025). This was a retrospective study of medical records involving minor patients. Data were accessed for research purposes between November 3 and November 15, 2025, following ethics approval. Written informed consent for the use of medical data for scientific and academic purposes is routinely obtained from parents or legal guardians at hospital admission, in accordance with institutional policy. The authors had access to identifiable patient information only during data extraction. All data were anonymized prior to analysis, and no identifiable information was accessible during or after data analysis. No additional procedures were performed for research purposes.

### Admission protocol

**Fig 1:**
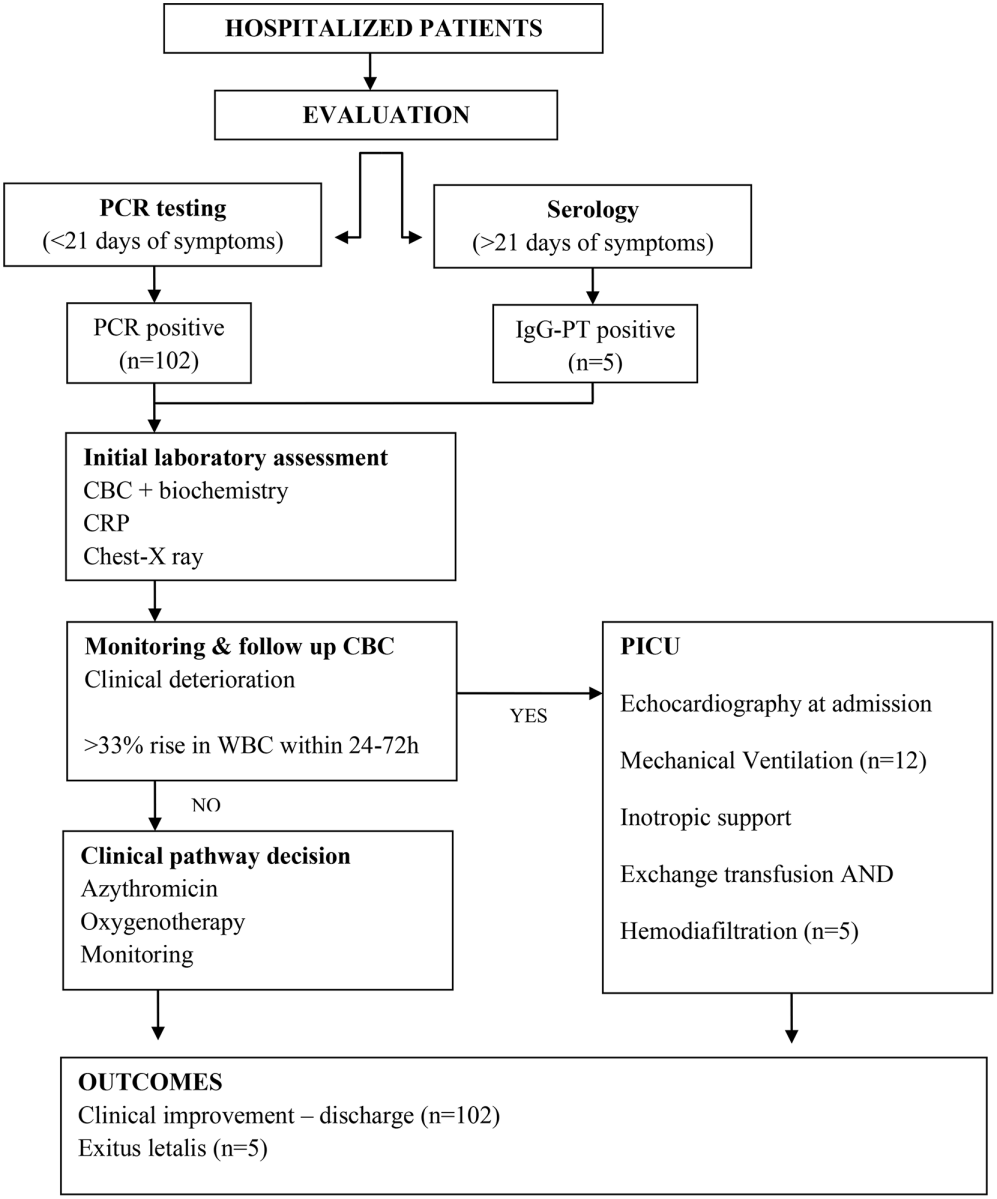
Flow diagram of patient selection, diagnostic evaluation, and clinical outcomes.

Upon admission, all patients underwent a standardized diagnostic protocol comprising routine laboratory testing—complete blood count, C-reactive protein, serum biochemistry, and urinalysis—alongside chest X-rays. To confirm clinical suspicion of pertussis, nasopharyngeal swabs were referred to the national reference laboratory for molecular analysis via polymerase chain reaction (PCR), while the anti-pertussis toxin IgG (IgG-PT) serological enzyme-linked immunosorbent assay (ELISA) was performed in patients evaluated more than three weeks after symptom onset, due to the possibility of false-negative PCR results (Fig 1). Serological positivity was defined based on elevated anti-pertussis toxin IgG levels, according to the reference laboratory standards and in line with established criteria, taking into account the timing of symptom onset and prior vaccination [12]. The laboratory provided qualitative interpretation (positive/negative) based on validated internal cut-offs.

Azithromycin was administered according to standard weight-based dosing guidelines unless previously initiated. Follow-up laboratory testing was performed within 24–72 hours after admission to assess leukocyte dynamics. Complete blood count was used both for diagnostic and monitoring purposes, with particular attention to rising leukocyte trends and their clinical correlation. Clinical deterioration in the presence of increasing leukocyte counts prompted closer monitoring and consideration of PICU transfer in accordance with institutional protocol.

### PICU admission criteria and interventions

Patients presenting with severe clinical and laboratory features—including respiratory distress, frequent apneas, hemodynamic instability, and altered consciousness—were admitted to the PICU. Laboratory criteria for escalation included marked leukocytosis or a sustained upward trend in leukocyte count, as previously defined. Upon PICU admission all patients underwent echocardiography to assess cardiac function and screen for pulmonary hypertension. Depending on individual clinical needs, patients received advanced supportive measures such as mechanical ventilation, inotropic stimulation, renal replacement therapy, or exchange transfusion.

Exchange transfusion was considered in cases of extreme leukocytosis. Although no single leukocyte threshold served as an absolute indication, the decision was individualized based on the patient’s clinical status, presence of pulmonary hypertension, and the trajectory of leukocyte elevation. In the most severe cases, where pronounced hyperleukocytosis persisted after EST and was accompanied by clinical and laboratory signs of systemic inflammation, continuous venovenous hemodiafiltration (CVVHDF) was employed as a sequential adjunctive therapy to further reduce leukocyte burden and inflammatory mediators.

### Discharge criteria

Patients who demonstrated clear clinical recovery—marked by resolution of respiratory symptoms and sustained afebrile status—alongside normalization of leukocyte count and CRP levels, were considered eligible for discharge. Once patients were clinically stable and laboratory parameters remained within normal limits over consecutive assessments, a 24–48 hour observation phase was initiated to confirm sustained recovery prior to discharge.

### Statistical analysis

Statistical analyses were performed using JASP (Version 0.95.3). Descriptive statistics were used to summarize demographic and clinical variables. For comparisons of independent groups, the Mann–Whitney U test was used for non-normally distributed continuous variables, and the Kruskal–Wallis test was applied when more than two groups were compared; where appropriate, Dunn’s post hoc analysis was conducted. The Wilcoxon signed-rank test was employed for paired comparisons of inflammatory parameters at admission and within the first 72 hours of hospitalization. Chi-square tests were used to analyze categorical variables, while binomial tests were applied to assess proportions against expected values. Binary logistic regression was performed to identify predictors of adverse clinical outcomes, including pneumonia, mechanical ventilation, PICU admission, and in-hospital mortality.

Variables with a univariate association at p < 0.2 were considered for inclusion in the multivariate logistic regression model, following the purposeful selection strategy described by Hosmer and Lemeshow [13]. This approach allows retention of potentially important predictors and confounders that may not appear significant in isolation. Final models were refined based on clinical relevance, statistical significance, and multicollinearity diagnostics (variance inflation factor < 2). Given the limited sample size and small number of outcome events, these analyses were considered exploratory and hypothesis-generating. Odds ratios (ORs) with 95% confidence intervals were reported.

## Results

A total of 107 pediatric patients diagnosed with pertussis were hospitalized during the study period; outpatient or non-hospitalized cases were not included in this analysis. Of these, the diagnosis was confirmed by PCR testing in 102 patients, whereas in 5 patients it was established serologically (Fig 1). The latter applied exclusively to fully vaccinated individuals who had experienced symptoms for more than three weeks.

There was no statistically significant difference in sex distribution (p > 0.05). The median age at admission was 2 months (IQR 1.5–4 months). Most patients were admitted within the first 10 weeks of life (65/107, 61%). The majority of patients were born at term; term births were significantly more frequent than preterm births (91 vs. 16, p < 0.01).

Regarding vaccination status, 27/107 (25%) patients had received at least one dose of pertussis vaccine, while 80/107 (75%) were completely unvaccinated. This difference was statistically significant (p < 0.001). Among the vaccinated subgroup, 15/27 (55.5%) had received only a single dose. Notably, among the 16 prematurely born patients, 13 were unvaccinated and 2 had received only a single dose (one was fully vaccinated for age), while there were only four children within the first ten weeks (4/65) that received the first vaccine dose.

Data on maternal pertussis vaccination during pregnancy were available; however, none of the mothers had received pertussis vaccination during pregnancy. Demographic characteristics are summarized in Table 1.

**Table 1:** Demographic and Characteristics of Cough and Associated Symptoms.

Parameter	Overall(n = 107)	Nonvaccinated (n = 80)	Partially vaccinated (n = 16)	Fully vaccinated(n = 11)
Sex (Male / Female)	52 / 55(48.6% / 51.4%)	40 / 40	6 / 10	6 / 5
Age at Admission(Median, IQR)	2 months(1.5–4 months)	1.75 months(1.37 – 2.50)	3 months(2.75–3.0)	6 years(2.41–10)
Gestational Age(Term / Preterm)	91 / 16(85% / 15%)	13/80 (16%)	2/16 (12.5%)	1/11 (9%)
Cough at admission	107 / 107 (100%)			
‣ Paroxysmal cough	88 / 107 (82.2%)	68/80 (85%)	14/16 (87%)	6/11 (54%)
‣ Non-paroxysmal (classic) cough	20 / 107 (17.8%)	12/80 (15%)	2/16 (13%)	5/11 (46%)
Median duration of coughbefore admission	10 days (IQR 5–14)	7 days(IQR 5–14)	10 days(IQR 5–14)	20 days(IQR 14–20)
Associated symptoms(vomiting, cyanosis, apnea)	66 / 107 (61.7%)	53/80 (66%)	8/16 (50%)	5/11 (45%)
‣ Cyanosis	55 / 107 (51.4%)	44/80 (55%)	8/16 (50%)	3/11 (27%)
‣ Vomiting	18 / 107 (16.8%)	14/80 (17.5%)	1/16 (6%)	3/11 (27%)
‣ Apneic episodes	24 / 107 (22.4%)	20/80 (25%)	1/16 (6%)	3/11 (27%)

*Overall P values are provided; detailed post‑hoc comparisons are described in the text.

Cough was the most frequent symptom at admission, present in all 107/107 (100%) patients. Paroxysmal cough was observed in a statistically significant majority (p < 0.001). In a substantial proportion of cases, cough was accompanied by at least one of the following symptoms: vomiting, cyanosis, or apneic episodes (66/107, 61.7%). Among these associated symptoms, cyanosis was the most common (55/107; 51.4%), while vomiting and apneic episodes were less common (Table 1).

The median duration of cough prior to admission was 10 days. Stratified by vaccination status, the median duration was 7 days in unvaccinated patients, 10 days in those who had received a single vaccine dose, and 20 days in patients considered fully vaccinated for age. The Kruskal–Wallis test demonstrated a statistically significant difference in cough duration across these groups (p = 0.010), with Dunn’s post hoc analysis confirming that the difference was particularly pronounced between unvaccinated and fully vaccinated patients (p < 0.008). The prevalence of paroxysmal cough differed across vaccination groups (87.5% in partly vaccinated, 85.0% in unvaccinated, and 54.5% in fully vaccinated patients, p = 0.039). However, this finding likely reflects the substantial differences in age distribution between groups, particularly the higher median age in the fully vaccinated group.

At admission, the median leukocyte count was 19.20 × 10⁹/L, with lymphocytes constituting the predominant fraction (median 12.27 × 10⁹/L). The median C-reactive protein (CRP) level was 0.3 mg/L, and the median absolute neutrophil count was 4.37 × 10⁹/L. No statistically significant differences were observed in leukocyte count, lymphocyte count, neutrophil count, or CRP levels with respect to sex, age, or gestational age at birth (Table 2).

**Table 2:** Laboratory Parameters and Radiographic Pneumonia at Admission.

Parameter	At admission	Within 72h of admission^1^	p-value
Leukocyte count (×10⁹/L)	19.20 (14.04–28.75)	27.34 (19.51-40.56)	0.568
Lymphocyte count (×10⁹/L)	12.27 (8.92–19.55)	19.80 (11.55-26.75)	0.724
Neutrophil count (×10⁹/L)	4.37 (2.92–7.16)	6.03 (3.74-10.36)	0.406
CRP (mg/L)	0.3 (0.1–1.2)	1.7 (0.25-24.55)	0.813
N/L ratio	0.34 (0.22-0.54)	0.39 (0.21-0.63)	0.537
Radiographically confirmed pneumonia	30/107 (28%)	—	—

*Values are presented as median (interquartile range, IQR); ^1^Follow-up laboratory data at 72 hours were not available for all patients.

Repeated laboratory analyses were available for 76/107 children (71%) within the first 72 hours. These showed an overall trend toward increasing inflammatory parameters. While this rise was evident in some individual patients, it did not reach statistical significance when assessed across the cohort as a whole (Table 2).

Radiographic findings consistent with pneumonia were identified in 30 of 107 patients (28%), while the majority showed no pathological changes on chest X-ray at admission (77/107; 72%). Leukocyte count, lymphocyte count, and CRP levels were significantly higher in patients with radiographic evidence of pneumonia compared to those without (p < 0.001, p = 0.004, and p < 0.001, respectively). In univariate analysis, radiographic evidence of pneumonia was significantly associated with severe outcomes, including the need for MV, pulmonary hypertension, EST and CVVHDF, and mortality (all p < 0.01). This variable was not included in the multivariable models to avoid multicollinearity with related predictors, particularly inflammatory markers.

Viral coinfection was documented by PCR in 24 of the 107 patients (22.4%). The most frequently identified pathogens were respiratory syncytial virus (RSV) in 11 cases (10.3%) and rhinovirus in 8 cases (7.5%). Less common coinfections included SARS-CoV-2 in 4 patients (3.7%), and single cases of influenza, adenovirus, enterovirus, and cytomegalovirus (each 0.9%). Among the 24 children with documented viral coinfection, five died (20.8%), whereas no deaths occurred among the 83 patients without coinfection. This association between viral coinfection and mortality was statistically significant (χ² test, p < 0.001).

Of the 107 children with pertussis, 13 (12.1%) required admission to the PICU and 12 (11.2%) underwent MV. Five patients underwent dialysis, three received exchange transfusion, and six developed pulmonary hypertension. The overall case fatality rate was 4.7% (5/107 children). Of the 13 infants admitted to the PICU, 12 were younger than two months of age, as were 11 of the 12 who required MV. Prematurity was notably overrepresented among the most severe cases: 5 of the 13 admitted to the PICU were preterm (p = 0.011), and 5 of the 12 who required mechanical ventilation were also preterm (p = 0.006). Importantly, all preterm infants were part of the < 2-month age group. All fatal cases occurred within the first two months of life.

### Multivariable logistic regression models

In the multivariable logistic regression model predicting radiographically confirmed pneumonia, which included prematurity, early age, absolute lymphocyte count (ABL), and neutrophil-to-lymphocyte (N/L) ratio, three predictors reached statistical significance. Prematurity was the strongest independent predictor (OR = 10.76, p = 0.002), although the estimate was associated with a wide confidence interval. Early age (defined as the first two months of life) was also significantly associated with increased odds of pneumonia (OR = 5.24, p = 0.015). In addition, a higher absolute lymphocyte count was associated with increased risk (OR = 1.08 per unit increase, p = 0.003). The N/L ratio showed a positive association with pneumonia; however, this did not reach statistical significance in the final model (OR = 2.09, p = 0.072). The model was statistically significant compared to the null (Δχ² = 26.53, p < 0.001). Pseudo-R^2^ measures (Nagelkerke R² = 0.345) are reported descriptively and should be interpreted with caution, particularly in small samples.

Logistic regression analysis demonstrated that the model predicting PICU admission was statistically significant (χ² = 27.77, p < .001). Pseudo-R² measures (Nagelkerke R² = 0.472, Tjur’s R² = 0.374) are reported descriptively and should be interpreted with caution, especially in small samples, as they are not directly comparable to R² from linear regression models. Prematurity emerged as the strongest independent predictor, although the effect estimate was associated with a wide confidence interval (Table 3). In addition, younger age, higher absolute lymphocyte count, and an elevated neutrophil-to-lymphocyte ratio upon admission were all significantly associated with PICU admission, supporting a possible contribution of early immune response dynamics to disease progression.

**Table 3:** Multivariable Logistic Regression Models for Adverse Outcomes in Children with Pertussis.

Predictor	PICU admission OR (95% CI), p	Mechanical ventilation OR (95% CI), p
Early age (<2 months)	25.06 (1.57–400.2), 0.016*	26.42 (1.91–365.3), 0.018*
Prematurity	20.63 (2.98–142.7), 0.002*	30.67 (3.66–256.9), 0.001*
Absolute lymphocyte count	1.10 (1.02–1.19), 0.009*	1.11 (1.03–1.21), 0.007*
N/L ratio	3.50 (1.11–11.0), 0.032*	4.16 (1.30–13.3), 0.018*
Viral coinfection	—	—

* Statistically significant predictors;

The model predicting the need for mechanical ventilation was also statistically significant (χ² = 29.56, p < .001; Nagelkerke R² = 0.518, Tjur’s R² = 0.415), although pseudo-R² values should be interpreted with caution, particularly in small samples. Prematurity, younger age, elevated lymphocyte count, and higher N/L ratio were all independently associated with an increased risk of mechanical ventilation, reflecting the combined impact of host vulnerability and inflammatory response.

Vaccination status was not included in the multivariable models due to its strong association with age and the resulting collinearity, which precluded reliable estimation of its independent effect.

Mortality occurred in 4.7% of the cohort. All fatal cases occurred in infants younger than two months of age who were unvaccinated and had documented viral coinfection. In exploratory analysis, the neutrophil-to-lymphocyte ratio was significantly higher in non-survivors compared to survivors (Mann–Whitney U = 74, p = 0.011), suggesting a potential role of immune dysregulation in fatal disease. Given the small number of fatal events, these findings should be interpreted with caution. Nevertheless, the observed pattern underscores the vulnerability of very young unvaccinated infants to severe pertussis and its complications.

## Discussion

In this study, we analyzed clinical and laboratory predictors of severe and fatal pertussis in a pediatric cohort. Although statistical power was limited by the small number of deaths, the pattern of outcomes was consistent: all fatal cases occurred in unvaccinated infants younger than two months, before they could receive their primary pertussis vaccination. This finding highlights the particular vulnerability of early infancy and suggests that the absence of maternal antibody protection may have contributed to the observed mortality. Together with the observed associations between viral coinfection, elevated N/L ratio, and fatal outcomes, these results emphasize the potential contribution of pathogen burden and immature host immunity in early-life pertussis. In line with previous reports, our findings support the importance of maternal immunization as a strategy to confer passive protection during the first months of life.

It has been suggested that among infants in the first weeks of life, those born preterm are particularly vulnerable to severe disease [1,8,9]. In our analysis, prematurity emerged as an independent predictor of both PICU admission and the need for mechanical ventilation, although the effect estimates were associated with wide confidence intervals. This finding reflects the particular vulnerability of preterm infants, whose pulmonary and immune systems are immature and less capable of mounting an effective response [9,14]. In addition, preterm infants are universally unvaccinated at the time of infection and receive lower levels of maternally transferred antibodies due to reduced transplacental transport in late gestation, further compounding their susceptibility [14]. In our cohort vaccination status was strongly associated with age, as older children were more likely to be fully immunized, whereas younger infants were predominantly unvaccinated due to their age. This introduces a high degree of collinearity between age and vaccination status, limiting their simultaneous inclusion in multivariable models and complicating the interpretation of vaccination effects. Accordingly, vaccination status was not included in the final regression models, and differences observed across vaccination groups should be interpreted with caution, as they are likely driven by age-related factors rather than representing an independent effect of vaccination. Although reduced passive antibody transfer limits the degree of protection that maternal vaccination can provide to premature infants, maternal immunization remains an important preventive measure, as it increases baseline maternal antibody titers and thereby maximizes the level of passive protection achievable even in early preterm deliveries [14]. Evidence from countries that have introduced routine maternal immunization demonstrates a substantial reduction in the burden of infant pertussis [15,16]. In contrast, maternal vaccination is not routinely implemented in our country, which may explain the high proportion of infants in the first ten weeks of life among hospitalized cases in our cohort. In line with this, none of the mothers in our cohort had received pertussis vaccination during pregnancy, further emphasizing the absence of maternally derived protection in early infancy. Taken together, these considerations suggest that redefining immunization strategies, with particular emphasis on both maternal and adolescent vaccination, may represent a critical step toward reducing the overall population burden of pertussis and protecting the most vulnerable groups.

Beyond demographic risk factors, laboratory markers provide additional insight into disease severity [11,17]. The prognostic value of WBC count and ABL in pertussis has long been recognized, both as initial laboratory markers and as indicators of disease progression [11,17]. While an elevated ABL is undoubtedly associated with severe clinical forms, accumulating evidence suggests that pertussis severity reflects a broader state of immune dysregulation rather than isolated lymphocytosis [8]. Increasing attention has been paid to indices derived from standard hematological parameters, particularly the N/L ratio [18]. Elevated neutrophil counts, an altered N/L ratio, and excessive cytokine activation appear to act synergistically, driving systemic endothelial injury and multiorgan dysfunction [1].

These pathophysiological changes likely reflect the impact of extreme leukocytosis and dysregulated inflammatory mediators on the vascular endothelium, leading to impaired microcirculatory flow, endothelial damage, and activation of the coagulation cascade [19]. Pulmonary vessels are particularly affected, and autopsy findings have consistently demonstrated leukocyte aggregates and endothelial injury in this setting [19]. Clinically, this mechanism explains the poor response to pulmonary vasodilators in critically ill patients, as pulmonary hypertension in pertussis does not arise from primary vasoconstriction but from impaired blood flow caused by cellular obstruction and vascular injury.

Although individual clinical and laboratory parameters may be useful, they are not always sufficiently specific or sensitive on their own. In this regard, multivariable regression analyses may help explore associations between multiple clinical and laboratory factors and severe disease outcomes [18]. In our study, the multivariable logistic regression models developed for PICU admission and the need for mechanical ventilation identified several factors associated with disease outcomes. However, these findings should be interpreted with caution, as the analyses were performed on a relatively small, single-center dataset without internal or external validation. Therefore, these models should be considered exploratory and hypothesis-generating rather than definitive predictive tools.

In addition to host immune dysregulation, co-pathogens may further amplify disease severity [5,20]. Respiratory viral infections are a well-recognized cause of acute bronchiolitis and pneumonia in infants, with respiratory syncytial virus (RSV) being the classic example. Their contribution to the severity of pertussis has also been highlighted in previous studies, and our findings provide additional support for this association [5,9,20,21]. Consistent with earlier reports, which showed that viral coinfection increases the likelihood of severe outcomes in infants with pertussis, our data suggest a similar pattern: children with documented viral coinfection had significantly higher rates of both PICU admission and mortality, supporting the potential relevance of viral coinfection in severe clinical forms of pertussis. The heterogeneity of viral agents identified in our study illustrates how easily the already fragile immune balance in infants with pertussis can be disrupted, tipping the course toward more severe disease. This aligns with published evidence showing that even non-RSV respiratory viruses may exacerbate pertussis severity, suggesting that the interaction between Bordetella pertussis and viral pathogens is likely driven more by host susceptibility than by virus-specific factors [9,20]. These observations emphasize the importance of considering viral coinfection not only as a complicating factor but also as a potential target for therapeutic intervention in the management of severe pertussis. Given the absence of specific antiviral therapies for viral pathogens, preventive strategies have become a focal point of pediatric infectious disease management. A notable example of an effective preventive approach is the use of long-acting monoclonal antibodies for RSV prophylaxis in infants, which are designed to confer passive immunity during periods of heightened susceptibility. This paradigm underscores the importance of early immunization as part of a comprehensive strategy to prevent infectious diseases, emphasizing that timely protection is crucial to reducing disease burden, complications, and mortality among this vulnerable population.

Therapeutic strategies aimed at leukoreduction have been explored but remain of uncertain benefit [6,11,22]. EST represents the most common intervention, and this procedure effectively lowers both leukocyte counts and circulating pertussis toxin. However, persistent elevation of inflammatory mediators remains a significant problem and may at least partly account for the frequent occurrence of secondary infections [11,23,24].

These observations suggest that EST alone only partially addresses the underlying pathophysiology. Indeed, a rebound increase in lymphocyte counts is often seen in the days following the procedure, and the mortality benefit reported in both our series and previous studies has been inconsistent [23–25]. This raises the question of whether EST should be combined with adjunctive approaches such as extracorporeal membrane oxygenation (ECMO) or CVVHDF, targeting not only leukocyte depletion but also the removal of pro-inflammatory mediators [22,26]. While ECMO was not available in our center until late 2024, CVVHDF was employed as an adjunctive measure in selected cases. Although this treatment modality has not previously been reported for pertussis and is not included in current treatment guidelines, its use was guided by experience in septic shock, where it is applied to remove excessive circulating cytokines [27]. Although our cohort was too small to draw firm conclusions, in four patients who underwent EST followed immediately by CVVHDF as a sequential strategy, two survived and two did not, despite the combined intervention. This suggests that the approach, while theoretically sound, is not universally effective. A plausible explanation is that there may be a narrow therapeutic window during which such interventions can meaningfully alter the disease trajectory; beyond this point, once multisystem complications are established, the benefits may be markedly reduced. Defining this window of opportunity, as well as the optimal sequencing of interventions, will require larger multicenter studies with harmonized protocols [26]. In this context, multivariable analyses identified several factors associated with PICU admission and the need for mechanical ventilation. However, these findings should be interpreted as exploratory, given the small sample size and the lack of internal and external validation. Prospective validation in independent cohorts is required before any clinical application can be considered. Predictive approaches based on routinely available clinical and laboratory data at admission may help to identify patterns associated with disease progression. Such approaches may help generate hypotheses for future multicenter studies evaluating disease progression and timing of escalation strategies in severe pertussis [28,29].

### Study limitations

Finally, several limitations of our study should be acknowledged. First, it was conducted in a single center with a relatively small number of patients with the most severe clinical forms, thus reducing the statistical power to detect certain associations. Second, the study covered a relatively long observation period, during which therapeutic protocols—particularly those concerning exchange transfusion and dialysis—were gradually adapted and refined, potentially introducing heterogeneity in management. Furthermore, admission timing, laboratory parameters, and treatment initiation varied across patients in relation to the onset of disease, reflecting considerable individual differences that may have influenced outcomes. The small number of severe outcome events, particularly mortality, PICU admission, and mechanical ventilation, resulted in unstable estimates and limited robustness of the multivariable analyses. These analyses should therefore be interpreted as exploratory and hypothesis-generating. Furthermore, no internal or external validation was performed. Finally, data on the broader epidemiological context, such as transmission patterns in maternity wards and within households, were lacking but would have provided valuable additional insights.

## Conclusions

Recent years have seen a resurgence of pertussis, with increasing outbreaks reported across multiple countries. Severe disease continues to disproportionately affect unvaccinated infants, who remain at highest risk for complications. In this cohort, severe and fatal outcomes were concentrated in the youngest patients, underscoring their vulnerability.

Although therapeutic options remain limited, interventions such as leukoreduction and modulation of the inflammatory response may be beneficial in selected cases and warrant further investigation.

These findings support the importance of preventive strategies targeting early infancy, including maternal vaccination, as a potential approach to reducing disease burden in this vulnerable population. Larger multicenter studies are needed to further evaluate factors associated with severe disease and to clarify optimal prevention and treatment strategies.

## Supporting information

S1 Data Study dataset. De-identified participant-level data used for the analyses.
(CSV)

S2 Data Variable definitions and coding scheme. Definitions and coding of all variables included in the S1 Dataset. (TXT)
