# Supplementary material for: Clinical features, disease severity, and outcomes of pertussis in hospitalized children: A retrospective study from Serbia
Source: PLoS One. 2026 Jun 26;21(6):e0352498. doi: 10.1371/journal.pone.0352498 (PMC13308863; doi:10.1371/journal.pone.0352498)
Supplement: Attachment — Submitted filename: Response to Reviewers.docx [file pone.0352498.s004.docx]

**Response to Reviewers**

Clinical features, disease severity, and outcomes of pertussis in hospitalized children: a retrospective study from Serbia

**REVIEWER 1**

**1)** The title is a little misleading and should be revised- this is not a report of an outbreak of pertussis or a vaccine policy review. The title should be revised, a more appropriate title could be 'Predictors of mortality and disease severity amongst children with pertussis in Serbia'

**Response:**

We thank the reviewer for this helpful suggestion. We agree that the original title may have overstated the scope of the study. In response, we have revised the title to more accurately reflect the study design and its primary focus on clinical characteristics, disease severity, and outcomes in hospitalized children with pertussis.

The revised title is: *“Clinical features, disease severity, and outcomes of pertussis in hospitalized children: a retrospective study from Serbia.”*

**2)** In the Introduction (line 87-89) the authors state 'the pertussis booster vaccination for pregnant women is recommended... although it is not included in the mandatory schedule'. This is confusing. Do the authors mean that pertussis vaccination for pregnant women is recommended but that uptake is low? Please clarify, and if data are available about vaccine uptake for pregnant women (and the general population) in Serbia, it would be important to include.

**Response:**

We thank the reviewer for this important comment and the opportunity to clarify this point. We agree that the original wording may have been unclear.

In the Republic of Serbia, pertussis vaccination during pregnancy is recommended by national expert bodies as part of an extended immunization strategy; however, it is not included in the mandatory national immunization program. This means that, although vaccination is advised, it is not legally required and is therefore not systematically implemented.

We have revised the Introduction to clarify this distinction and improve readability.

**3)** The introduction should be strengthened to include references and additional context regarding recent concerning increases in pertussis cases and outbreaks in the US/Europe and as note by the WHO and also concerning declines in vaccination rates in some countries (see this article for a relevant summary- Vargas-Zambrano JC, Abrudan S, Macina D. Understanding the Epidemiology and Contributing Factors of Post-COVID-19 Pertussis Outbreaks: A Narrative Review. Infect Dis Ther. 2026 Jan;15(1):19-41. doi: 10.1007/s40121-025-01277-1. Epub 2025 Dec 1. PMID: 41324875; PMCID: PMC12783469.)

**Response:**

We thank the reviewer for this valuable suggestion. We agree that additional context regarding recent global trends in pertussis epidemiology strengthens the Introduction.

In response, we have expanded the Introduction to include recent evidence on the resurgence of pertussis in the post-COVID-19 period, including reports from Europe and North America, as well as contributing factors such as declining vaccination coverage, waning immunity, and insufficient uptake of booster doses.

The Introduction now includes the following statement:

“*In recent years, a resurgence of pertussis has been observed globally, particularly in the post-COVID-19 period, with outbreaks reported across Europe and North America…*”

We have also incorporated the suggested reference (Vargas-Zambrano et al., 2026) to support this context.

**4)** Methods- line 113-115- please clarify whether the patients included were consecutive patients with pertussis, all patients between the dates mentioned, or were selected in another way (e.g. at random)? How were eligible patients with pertussis identified (e.g. via PCR/serology results, or via a case registry, or via discharge diagnosis data?)?

**Response:**

We thank the reviewer for this helpful comment. We agree that clarification of the patient selection process improves methodological transparency.

In response, we have revised the Methods section to specify that all eligible hospitalized patients during the study period were consecutively included and identified through hospital medical records based on confirmed pertussis diagnosis. The following sentence has been added:

“*All eligible patients hospitalized during the study period were consecutively included. Patients were identified through hospital medical records based on confirmed pertussis diagnosis.*”

**5)** Methods- please describe how positive serology was defined (especially for those who had received vaccination previously) and what serologic test was used.

**Response:**

We thank the reviewer for this important comment. We agree that clarification of the serological criteria is essential.

In our study, serological testing was performed in a national reference laboratory using anti-pertussis toxin IgG (IgG-PT) ELISA. Serological positivity was defined based on elevated IgG-PT levels according to the reference laboratory standards, in line with established criteria (Cherry et al., 2012), and interpreted in the context of symptom duration and prior vaccination. The laboratory provided qualitative interpretation (positive/negative) based on validated internal cut-offs.

This clarification has now been added to the Methods section:

“*Serological positivity was defined based on elevated anti-pertussis toxin IgG levels, according to the reference laboratory standards and in line with established criteria, taking into account the timing of symptom onset and prior vaccination (12). The laboratory provided qualitative interpretation (positive/negative) based on validated internal cut-offs.*”

**6)** Methods- please given a definition for fully/partially vaccinated in the methods, prior to discussing the results for this metric in line 210. Please also give a clear definition for prematurity.

**Response:**

We thank the reviewer for this helpful comment. We agree that clear definitions of key variables improve the clarity of the Methods section.

In response, we have revised the Methods to explicitly define prematurity and vaccination status categories based on age-appropriate doses according to the national immunization schedule. The text now reads:

“*Prematurity was defined as birth before 37 completed weeks of gestation. Vaccination status was categorized as follows: children were considered unvaccinated if they had not received any pertussis-containing vaccine; partially vaccinated if they had received at least one but not all age-appropriate doses; and fully vaccinated if they had received all doses appropriate for their age according to the national immunization schedule.*”

**7)** Results- the first paragraph is

**Response:**

We thank the reviewer for this comment. As the specific concern regarding the first paragraph of the Results section was not clearly specified, we have carefully reviewed this section and made minor revisions to improve clarity and readability.

**8)** Results- Table 1 includes vaccination status or the infant/child. Was data regarding maternal vaccination status (i.e. pertussis booster during pregnancy) available? If yes, please include this. If no, then please add this to the discussion as a major limitation of the study

**Response:**

We thank the reviewer for this important comment. Data on maternal pertussis vaccination status were available for our cohort, and this information has now been explicitly included in the manuscript.

In the Results section, we have added the following statement:

“*Data on maternal pertussis vaccination during pregnancy were available; however, none of the mothers had received pertussis vaccination during pregnancy.*”

This finding has also been incorporated into the Discussion, where it is contextualized within the current national setting. The text now states:

“*In line with this, none of the mothers in our cohort had received pertussis vaccination during pregnancy, further emphasizing the absence of maternally derived protection in early infancy.*”

These additions clarify the maternal immunization status in our cohort and further support the interpretation of increased vulnerability among young infants.

**9)** Results- line 214- it is not clear what is statistically different here and what the authors mean by 'favoring term births'. Do the authors mean that severe pertussis cases or deaths were significantly frequent among pre-term infants? Please clarify.

**Response:**

We thank the reviewer for this helpful comment. We agree that the original wording may have been unclear.

In response, we have revised the sentence to explicitly state that term births were significantly more frequent than preterm births and to provide the corresponding numbers for clarity. The text now reads:

“*The majority of patients were born at term; term births were significantly more frequent than preterm births (91 vs. 16, p < 0.01).*”

**10)** Results- line 217-218- 'The proportion of unvaccinated patients was significantly higher'- I am not sure what the comparator here is. Please clarify. Do you mean that among severe case of pertussis the proportion unvaccinated was significantly higher?

**Response:**

We thank the reviewer for this helpful comment. We agree that the comparator was not clearly specified in the original wording.

In response, we have revised the sentence to clarify that the proportion of unvaccinated patients was compared to those who had received at least one vaccine dose. The text now reads:

“*Regarding vaccination status, 27/107 (25%) patients had received at least one dose of pertussis vaccine, while 80/107 (75%) were completely unvaccinated. This difference was statistically significant (p < 0.001).*”

**11)** Results- line 228- I am not clear what the p-value of 0.016 refers to. Please clarify. Was apnea significantly more likely than the vomiting or cyanosis, or was it that a significant majority of cases had at least one additional symptom?

**Response:**

We thank the reviewer for this helpful comment. We agree that the original wording did not clearly specify the comparison underlying the reported p-value.

Upon re-evaluation, we recognized that the p-value was not essential, as the statement is descriptive in nature. Therefore, we have revised the sentence to report the proportion of patients with accompanying symptoms without inferential testing, improving clarity and consistency with the data presented in Table 1. The text now reads:

“*In a substantial proportion of cases, cough was accompanied by at least one of the following symptoms: vomiting, cyanosis, or apneic episodes (66/107, 61.7%).*”

**12)** Discussion- if data about maternal pertussis vaccine booster during pregnancy was not available, please add this to the section discussing Study Limitations.

**Response:**

We thank the reviewer for this helpful comment. Data on maternal pertussis vaccination during pregnancy were available for our cohort and have now been explicitly reported in the Results section, showing that none of the mothers had received pertussis vaccination during pregnancy.

This finding has also been incorporated into the Discussion (as detailed in response to comment 8) and therefore does not represent missing data or a study limitation.

**13)** Conclusion- the authors state that 'pertussis outbreaks can still occur', but actually data from WHO and others (see the paper referenced above by Vargas-Zambrano et al) note worrying trends of increased outbreaks/cases in recent years. I recommend re-framing the beginning of the conclusion to remind the reader of the increased public health concern regarding rising pertussis outbreaks/cases in recent years across multiple countries.

**Response:**

We thank the reviewer for this valuable suggestion. We agree that the conclusion should better reflect the current global epidemiological context of pertussis.

In response, we have revised the opening of the Conclusion to emphasize the recent resurgence of pertussis and the increasing number of outbreaks reported across multiple countries, in line with recent literature (including Vargas-Zambrano et al., 2026).

The revised text now reads:

“Recent years have seen a resurgence of pertussis, with increasing outbreaks reported across multiple countries. Severe disease continues to disproportionately affect unvaccinated infants, who remain at highest risk for complications.”

Minor suggestions:

1) Suggest using MV as abbreviation for mechanical ventilation instead of IMV to improve readability.

**Response:**

We thank the reviewer for this helpful suggestion. In response, we have replaced the abbreviation “IMV” with “MV” throughout the manuscript to improve readability.

2) The methods section devotes a lot of space to describing the protocol for routine clinic care for pertussis at the center conducting the study (lines 118-183). This could be abbreviated/summarized.

**Response:**

We thank the reviewer for this helpful comment. We agree that the description of the clinical management protocol could be streamlined.

In response, we have revised and abbreviated this section by removing detailed procedural descriptions and focusing on the key elements relevant to the study design, while retaining sufficient information to provide appropriate clinical context.

3) Consider removing the p-values from Table 1- I am not sure they are very meaningful, except for the duration of cough differences which are already explored in the main results section text.

**Response:**

We thank the reviewer for this helpful suggestion. We agree that p-values in Table 1 may not be particularly informative, as the table is primarily descriptive in nature.

In response, we have removed p-values from Table 1 to improve clarity and avoid overinterpretation. Key differences are described and interpreted in the main Results section where appropriate.

4) Table 2- please state whether the numbers in brackets are the range or interquartile range for each parameter.

**Response:**

We thank the reviewer for this helpful comment. We agree that clarification of the values presented in Table 2 is important.

In response, we have added a table footnote specifying that all values are presented as median and interquartile range (IQR).

5) Results- Line 213- there is a typo and 'first two 10 weeks' should read 'first 10 weeks' I think.

**Response:** The text has been corrected accordingly to read “first 10 weeks.”

6) Please capitalize the 'f' in figure throughout where you are referring to a specific figure in the manuscript e.g. in line 320.

**Response:**

In response, we have standardized the formatting throughout the manuscript and capitalized “Figure” wherever it refers to a specific figure.

**REVIEWER 2**

**1.** At present, there are some obvious inconsistencies that need correction before the findings can be interpreted with confidence. The most striking example is in Table 1, where the vomiting row is not plausible as presented: the overall number is given as 18/107, but the subgroup values are listed as 66/80, 15/16, and 8/11. This cannot be correct and suggests either row displacement or transcription error. Similarly, Table 2 contains values that appear internally impossible, for example a leukocyte median of 27.34 with a range of 8.22–19.55. The same concern applies to other laboratory values in that table. These are not minor formatting issues, because they directly affect trust in the dataset and in the subsequent analyses. Please re-check all tables, denominators, ranges, and corresponding narrative statements very carefully.

**Response:**
 We thank the reviewer for carefully examining the tables and identifying these inconsistencies. We appreciate the reviewer’s attention to detail, which helped us improve the accuracy of the presented data.

In Table 1, the values in the row corresponding to vomiting were incorrectly reported due to a data entry error, where the number of patients without vomiting was inadvertently presented instead of those with vomiting. This has now been corrected, and the table reflects the accurate values.

In Table 2, the discrepancy in leukocyte values was due to incorrect reporting of the interquartile range. The median leukocyte count (27.34) was correct, while the interquartile range has now been corrected to 19.51–40.56.

In addition, all tables and corresponding values in the manuscript have been thoroughly rechecked against the original dataset, and any inconsistencies have been corrected. No further discrepancies were identified. These revisions do not affect the overall results or conclusions of the study.

These corrections improve data consistency and transparency without altering the interpretation of the results.

**2.** I understand the intention to identify early predictors of deterioration, and this is clinically worthwhile. However, the number of severe outcomes is very small: 13 PICU admissions, 12 cases requiring invasive mechanical ventilation, and 5 deaths. With events this limited, the models become very unstable, especially when multiple candidate predictors are entered. This instability is visible in Table 3, where some odds ratios are extremely large and have very wide confidence intervals. For mortality in particular, the estimates for N/L ratio and coinfection are extraordinarily imprecise, which suggests sparse-data bias and overfitting rather than robust effect estimation. The use of Firth’s method is sensible, but it does not fully solve the underlying limitation of very few outcome events. I would strongly encourage the authors to simplify the models, reduce the number of predictors, and frame these analyses much more cautiously as exploratory rather than definitive.

**Response:**
We thank the reviewer for this important and constructive comment. We fully agree that the limited number of adverse events—particularly for mortality—introduces a substantial risk of model instability, sparse-data bias, and overfitting when multiple predictors are included.

In response, we have simplified the multivariable logistic regression models by reducing the number of predictors and retaining only clinically relevant variables selected based on univariate screening and biological plausibility. The revised models for PICU admission and mechanical ventilation now include a reduced and consistent set of predictors, resulting in more stable and interpretable effect estimates with narrower confidence intervals. These changes are reflected in the revised Table 3, which now presents more stable and interpretable estimates.

The mortality analysis has been comprehensively revised in line with the reviewer’s concerns. Given the very small number of fatal events (n = 5) and the presence of complete separation (no deaths in the non-coinfection group), we avoided relying on multivariable regression as the primary analytical approach. Instead, we adopted a more transparent and statistically appropriate strategy:

– Viral coinfection was analyzed using a contingency table approach with a continuity correction, allowing estimation of the odds ratio despite zero events in one group.
 – The neutrophil-to-lymphocyte (N/L) ratio was assessed using a non-parametric comparison (Mann–Whitney U test), which demonstrated significantly higher values in non-survivors.

Firth’s penalized logistic regression was retained as an exploratory supplementary method to address separation; however, in accordance with the reviewer’s recommendation, its results are no longer presented as primary evidence. Instead, it is now described as yielding directionally consistent but imprecise estimates, and its limitations are explicitly acknowledged in the manuscript. This approach was chosen to provide more robust and transparent estimates given the limited number of events.

Accordingly, the Results section has been revised to reflect these methodological changes and to present a more cautious interpretation of the findings.

In addition, we have revised the Abstract and Discussion to ensure that all findings are consistently framed as exploratory and hypothesis-generating rather than definitive, in line with the limitations imposed by the small number of outcome events.

**3.** Some interpretations of the regression findings do not match the reported effect estimates. This needs close checking throughout. For example, in the pneumonia model, the manuscript states that a higher N/L ratio predicted higher odds of pneumonia, but the reported odds ratio is 0.66. An odds ratio below 1 indicates lower odds, not higher odds. This is important because readers will follow the narrative interpretation, and at the moment there is a mismatch between the statistical output and the wording used to explain it. I would recommend a line-by-line check of every regression statement in the Results and Discussion sections against the actual coefficients and direction of association in the table.

**Response:**
We thank the reviewer for carefully identifying this important inconsistency. We agree that the previously reported interpretation of the N/L ratio was incorrect, as the direction of the effect did not correspond to the reported odds ratio. This has now been corrected.

In addition, this section has been fully revised. The multivariable model has been simplified by reducing the number of predictors, in line with the reviewer’s recommendations and to ensure consistency with the models used for PICU admission and mechanical ventilation.

The revised model now includes a smaller set of clinically relevant variables, and all reported effect estimates and their interpretations have been carefully rechecked and aligned with the corresponding odds ratios.

The Results section has been updated accordingly. The revised text now reads:
“In the multivariable logistic regression model predicting radiographically confirmed pneumonia, which included prematurity, early age, absolute lymphocyte count (ABL), and neutrophil-to-lymphocyte (N/L) ratio, three predictors reached statistical significance. Prematurity was the strongest independent predictor (OR = 10.76, p = 0.002). Early age (defined as the first two months of life) was also significantly associated with increased odds of pneumonia (OR = 5.24, p = 0.015). In addition, a higher absolute lymphocyte count was associated with increased risk (OR = 1.08 per unit increase, p = 0.003). The N/L ratio showed a positive association with pneumonia; however, this did not reach statistical significance in the final model (OR = 2.09, p = 0.072).The model was statistically significant compared to the null (Δχ² = 26.53, p < 0.001) and demonstrated moderate explanatory power (Nagelkerke R² = 0.345).”

Overall, the interpretation of all regression findings is now fully consistent with the statistical output presented in the manuscript.

**4.** The claims regarding model performance are too strong for a small retrospective single-centre cohort. The manuscript presents the PICU and IMV models as having excellent or outstanding discrimination, based on AUC values of 0.951 and 0.987, and goes on to describe this as confirmation of model robustness. I think that language is too strong. These performance estimates appear to come from the same dataset used to build the models, and there is no internal validation reported. In a small dataset with very few events, apparent AUC values are often optimistic. Without bootstrapping, cross-validation, or external validation, these models should not be described as robust predictive tools. The results are interesting and hypothesis-generating, but they need a much more restrained interpretation.

**Response:**
We thank the reviewer for this important and insightful comment. We fully agree that the original wording overstated the performance and robustness of the predictive models, particularly in the context of a relatively small, single-center dataset without internal or external validation.

In response, we have revised both the Results and Discussion sections to provide a more cautious and appropriate interpretation of model performance.

In the Results section, we have recalculated model performance after simplifying the multivariable models and now report more conservative AUC values. The text has been revised as follows:

“The model predicting PICU admission demonstrated good apparent discrimination (AUC = 0.896), although this estimate should be interpreted with caution given that it was derived from the same dataset used for model development.

The model predicting the need for mechanical ventilation also showed high apparent discrimination (AUC = 0.921); however, no internal or external validation was performed, and these values may therefore overestimate true model performance.”

In the Discussion section, we have removed previously overstated expressions such as “excellent discrimination,” “outstanding performance,” and “confirmation of model robustness.” These have been replaced with a more balanced interpretation emphasizing the exploratory nature of the findings. Specifically, we have added the following statement:
“The multivariable logistic regression models developed for PICU admission and the need for mechanical ventilation demonstrated good apparent discriminative ability. However, these findings should be interpreted with caution, as the analyses were performed on a relatively small, single-center dataset without internal or external validation, which may result in optimistic estimates of model performance. Therefore, these models should be considered exploratory and hypothesis-generating rather than definitive predictive tools.”

These revisions align the interpretation of our findings with the limitations of the study design and address the reviewer’s concern regarding potential overstatement of model performance.

**5.** The title and policy framing should be moderated. The manuscript is based on a retrospective cohort of hospitalised children from a single tertiary centre. That design can certainly provide useful clinical and epidemiological insight, but it does not directly evaluate national vaccination policy, programme effectiveness, maternal vaccine uptake, or population-level prevention strategies. The current title asks whether it is time to change national vaccination policy, and the conclusion states that redefining national immunisation strategies may be the most effective means of protecting vulnerable age groups. I understand why the authors are making this connection, especially given that all fatal cases occurred in infants too young to be vaccinated. However, the study itself provides supportive observational evidence rather than direct policy evaluation. I think the paper would be stronger if the policy implications were framed more cautiously, as one reasonable interpretation of the observed vulnerability in early infancy rather than as a conclusion that follows directly from the data.

**Response:**
 We thank the reviewer for this important and constructive comment. We agree that the original title placed excessive emphasis on vaccination policy implications that are not directly evaluated within the scope of this retrospective single-center study.

In response, we have revised the title to more accurately reflect the study design and primary findings, focusing on clinical characteristics, disease severity, and outcomes in hospitalized children with pertussis.

The revised title is:

“Clinical features, disease severity, and outcomes of pertussis in hospitalized children: a retrospective study from Serbia.”

We have also moderated the policy-related statements in the Discussion and Conclusion to ensure that preventive implications are presented more cautiously and in line with the observational nature of the data, rather than as direct policy recommendations. This revision ensures better alignment between the title and the actual scope of the study.

**6.** The study population needs clearer description, particularly in relation to age and vaccination status. The manuscript repeatedly frames the cohort as “infants and young children”, and the median age overall is 2 months. However, Table 1 shows that the fully vaccinated group had a median age of 6 years. This means that the vaccination groups are clearly not age-comparable, which is expected in pertussis, but it makes group comparisons difficult to interpret. For example, differences in cough duration and symptom profile across vaccination groups may simply reflect age structure rather than vaccine effect. This needs to be acknowledged more explicitly, and the manuscript should avoid overinterpreting group differences where age is such a strong confounder.

**Response:**

We thank the reviewer for this important observation. We fully agree that vaccination status in our cohort is strongly confounded by age, as older children were more likely to be fully immunized, whereas younger infants were predominantly unvaccinated due to their age.

In response, we have revised the manuscript to more clearly describe the age distribution across vaccination groups and to explicitly acknowledge this limitation. We now emphasize that comparisons between vaccination groups should be interpreted with caution, as observed differences may largely reflect underlying age differences rather than a direct effect of vaccination.

In the Results section, we have clarified the handling of vaccination status in the multivariable analysis. The text now states:

“Vaccination status was not included in the multivariable models due to its strong association with age and the resulting collinearity, which precluded reliable estimation of its independent effect.”

In the Discussion, we have expanded this point by explaining the implications of this collinearity and its impact on interpretation, emphasizing that differences across vaccination groups should be interpreted cautiously and in the context of age as a major confounding factor. This clarification improves the internal validity of the interpretation of vaccination-related findings.

**7.** The ethics and consent description needs clarification. The manuscript states that this was a retrospective review of records from 2015 to 2025, approved in November 2025, and that written informed consent for the use of medical data was obtained from parents or legal guardians in accordance with institutional policy. This may be entirely appropriate locally, but as written it raises questions for the reader because retrospective chart reviews often rely on waiver or institutional approval for secondary use rather than fresh written consent. Please clarify how consent operated in practice here. Was consent already part of institutional admission procedures, or was there a specific ethics process for retrospective use of the data? This should be made fully transparent.

**Response:**

We thank the reviewer for this important comment and the opportunity to clarify this aspect of the study. Written informed consent for the use of medical data for scientific and academic purposes is routinely obtained from parents or legal guardians as part of standard institutional admission procedures.

To improve clarity, we have revised the Ethics statement in the Methods section to explicitly reflect this. The relevant sentence has been updated as follows:

“Written informed consent for the use of medical data for scientific and academic purposes is routinely obtained from parents or legal guardians at hospital admission, in accordance with institutional policy.”

This clarification has been incorporated to ensure full transparency regarding consent procedures.
