# Supplementary material for: Clinical features, disease severity, and outcomes of pertussis in hospitalized children: A retrospective study from Serbia
Source: PLoS One. 2026 Jun 26;21(6):e0352498. doi: 10.1371/journal.pone.0352498 (PMC13308863; doi:10.1371/journal.pone.0352498)
Supplement: Attachment — Submitted filename: Response to the reviewers - round 2.docx [file pone.0352498.s005.docx]

**Response to the reviewers**

We thank the reviewers for the careful evaluation of our manuscript and for the constructive comments. We have revised the manuscript accordingly and addressed all points raised. All changes are reflected in the revised version of the manuscript.

Reviewer #2: The authors have responded constructively to the previous round of review and the manuscript is substantially improved. The core findings are clinically meaningful and the analytical concerns raised by Reviewer 2 have been addressed in good faith. The following comments should be addressed before acceptance.

**1-** The Discussion still contains clinical utility language suggesting the models "may assist clinicians in identifying patients" and "guiding early management decisions." Given that no internal or external validation has been performed, this should be softened. Add a clear statement that prospective validation is required before any clinical application can be considered.

**Response:** Thank you for this comment. We agree that the clinical utility of the predictive models should be further tempered given the lack of internal and external validation. We have revised the Discussion to remove language implying clinical application and added a clear statement that prospective validation in independent cohorts is required before any clinical use can be considered. The relevant sentences have been rephrased accordingly.

**2-** The odds ratio for viral coinfection and mortality (47.1, 95% CI 2.5–888.1) is reported in Table 3 with a dash in place of a p-value, and no explanation for its absence. Either provide the p-value or add a brief note clarifying why it is not reported (for example, that Firth's penalized regression was used and conventional p-values are not applicable in this context).

**Response:** We thank the reviewer for this comment. Given the use of penalized regression, we focused on reporting effect estimates and confidence intervals, which are considered more informative in this setting. We have clarified this in the revised manuscript by adding an explanatory footnote to Table 3 and adjusting the table formatting accordingly.

**3-** The CI width for the coinfection-mortality association (2.5 to 888.1) means the data provide almost no information about the true effect size. Describing this finding as "exploratory" is not sufficient. The authors should state plainly that this estimate is statistically uninformative and should not be interpreted as evidence of a strong effect.

**Response:** We thank the reviewer for this important comment. We agree that the very wide confidence interval reflects substantial imprecision due to the small number of events. In the revised manuscript, we have explicitly clarified that the estimated odds ratio should not be interpreted as evidence of a strong effect and emphasized the limited robustness of these findings. The text has been revised accordingly.

**4-** Table 1: confirm that the corrected vomiting row values (18/107 overall, 14/80, 1/16, 3/11) are accurate and consistent with the dataset. The subgroup values now sum correctly, but this should be verified against the source data given prior transcription errors in this table.

**Response:** We thank the reviewer for this comment. We confirm that the corrected vomiting row values are accurate and consistent with the dataset. The previous issue resulted from a coding error in the initial table version, which has now been corrected and verified against the source data.

**5-** Table 2: the corrected leukocyte IQR within 72 hours (19.51–40.56) is now plausible against the reported median of 27.34. However, it would be worth adding a footnote clarifying that follow-up measurements were not available for all patients, if that is the case, to avoid readers assuming complete data.

**Response:** Thank you for this helpful comment. We have clarified that follow-up laboratory measurements within 72 hours were not available for all patients. Specifically, we have added a statement in the Results section indicating the number of patients with available follow-up data (76/107), and included a corresponding footnote in Table 2 to indicate incomplete data availability.

**6-** The sentence in the Results reporting the PICU model fit (Nagelkerke R² = 0.472) and the MV model (Nagelkerke R² = 0.518) should be accompanied by a brief acknowledgement that pseudo-R² values in logistic regression are not directly comparable to R² in linear models and should be interpreted with caution, particularly in small samples.

**Response:** We thank the reviewer for this important comment. In response, we have revised the Results section to include a statement clarifying that pseudo-R² measures in logistic regression are not directly comparable to R² from linear regression models and should be interpreted with caution, especially in the context of small sample sizes.

**7-** The authors note that vaccination status was excluded from multivariable models due to collinearity with age, which is the correct decision. However, the Results section still includes a comparison of paroxysmal cough prevalence across vaccination groups (85% unvaccinated vs 54.5% fully vaccinated, p=0.039) and interprets this as a vaccine effect. Given the median age of 6 years in the fully vaccinated group versus 1.75 months in the unvaccinated group, this difference almost certainly reflects age rather than vaccination. The interpretation should be revised accordingly.

**Response:** Thank you for this important comment. We agree that the observed difference in paroxysmal cough prevalence across vaccination groups is confounded by substantial differences in age distribution between groups. We have revised the Results section to remove any causal interpretation and to clarify that this finding most likely reflects age differences rather than an independent effect of vaccination.

**8-** The Acknowledgements disclose the use of QuillBot Premium for language editing. Authors should confirm that this use is consistent with PLOS ONE's policy on AI-assisted writing tools, and if required, provide clarification on the extent of its use.

**Response:** We thank the reviewer for this observation. We confirm that the use of QuillBot Premium complies with the policy of PLOS ONE regarding AI-assisted writing tools. The tool was used solely for linguistic refinement and did not contribute to the generation or interpretation of scientific content. The Acknowledgements section has been revised accordingly. The authors retain full responsibility for the integrity and accuracy of the work.

**9-** Minor: "Figure" should be capitalised consistently throughout the manuscript when referring to a specific figure. A few instances remain where it appears in lowercase.

**Response:** We thank the reviewer for this comment. We have carefully reviewed the manuscript to ensure consistent capitalization of the term “Figure” when referring to specific figures, and have confirmed that it is used consistently throughout the text.
