# Supplementary material for: Clinical features, disease severity, and outcomes of pertussis in hospitalized children: A retrospective study from Serbia
Source: PLoS One. 2026 Jun 26;21(6):e0352498. doi: 10.1371/journal.pone.0352498 (PMC13308863; doi:10.1371/journal.pone.0352498)
Supplement: Attachment — Submitted filename: Response to the reviewer.docx [file pone.0352498.s006.docx]

**Reviewer #2:** The authors have responded constructively to the previous review, and the manuscript has improved. The clinical dataset is valuable, particularly as a single-centre retrospective account of severe pertussis in hospitalised children in Serbia. However, several issues still require revision before the manuscript is ready for publication.

**1-** On mortality analysis. The mortality analysis remains the main concern. There were only five deaths, all occurring in unvaccinated infants younger than two months, and all with documented viral coinfection. The reported odds ratio for viral coinfection and mortality, 47.1 with a 95% CI of 2.5 to 888.1, is too imprecise to be clinically or statistically informative. I recommend removing this estimate from Table 3 and presenting the mortality findings descriptively in the Results. The descriptive pattern is itself important and does not require regression modelling.

**Response:** We thank the reviewer for this important comment. We agree that the mortality analysis was limited by the very small number of fatal events, resulting in unstable and imprecise regression estimates. In response, we removed the mortality regression estimates from Table 3 and revised the corresponding Results section to present mortality findings descriptively rather than through regression modelling. Specifically, references to continuity-corrected odds ratios and penalized logistic regression were removed. The revised text now emphasizes the observed clinical pattern and exploratory findings without suggesting reliable effect estimation.

**2-** On AUC and ROC analysis. The reported AUC values for PICU admission and mechanical ventilation are apparent AUCs calculated from the same dataset used to develop the models. No internal or external validation has been performed. These values may therefore overestimate model performance. I recommend either performing internal validation, for example using bootstrap optimism correction, or removing the AUC values and ROC figure. Given the sample size and event numbers, removing them and reframing the models as exploratory association analyses would be the more appropriate option.

**Response:** We thank the reviewer for this important comment. We agree that the reported AUC values represented apparent performance estimates derived from the same dataset used for model development and therefore could overestimate true model performance. Given the limited sample size and number of outcome events, we decided not to pursue formal internal validation. Instead, in accordance with the reviewer’s recommendation, we removed the ROC analyses, AUC values, and Figure 2 from the manuscript and reframed the multivariable analyses as exploratory association analyses rather than predictive models.

**3-** On prediction-model framing. The manuscript still gives the impression that the models have predictive or clinical risk-stratification value. This should be softened throughout. The models should be described as exploratory and hypothesis-generating only. Any wording implying clinical use, early identification for practice, or management guidance should be removed unless the models are properly validated.

**Response:** We thank the reviewer for this important comment. In response, we revised the manuscript throughout to soften predictive framing and to emphasize the exploratory nature of the multivariable analyses. References implying clinical applicability, risk stratification, or management guidance were removed or rephrased. The models are now consistently described as exploratory and hypothesis-generating, and the corresponding sections in the Statistical Analysis, Results, Discussion, and Conclusions were revised accordingly.

**4-** On number of outcome events. The models for PICU admission and mechanical ventilation are based on small numbers of events: 13 PICU admissions and 12 cases requiring mechanical ventilation. This creates a risk of overfitting and unstable estimates. This limitation should be stated more clearly in the Results and Discussion.

**Response:** We thank the reviewer for this important observation. We agree that the relatively limited number of severe outcome events, particularly PICU admission and mechanical ventilation, increases the risk of overfitting and unstable estimates. In response, we added explicit statements in both the Results and Discussion sections emphasizing that the multivariable analyses should be interpreted cautiously and considered exploratory and hypothesis-generating.

**5-** On prematurity estimates. The associations between prematurity and severe outcomes are clinically plausible, but the confidence intervals are very wide. For example, the estimates for PICU admission and mechanical ventilation span more than an order of magnitude. The authors should avoid presenting the point estimates as precise. Phrases such as “more than a 20-fold increase in risk” should be qualified or revised.

**Response:** We thank the reviewer for this valuable comment. We agree that the confidence intervals surrounding the prematurity estimates were wide and that the point estimates should not be interpreted as precise measures of effect size. Accordingly, we revised the Results and Discussion sections to remove wording suggesting precise magnitude of risk and added clarifying statements emphasizing the imprecision and cautious interpretation of these estimates.

**6-** Table 3 should be revised. I recommend removing the mortality column or limiting it to descriptive information. If the authors retain the table, it should not present unstable mortality estimates in a way that suggests reliable inference.

**Response:** We thank the reviewer for this helpful suggestion. Table 3 has been revised accordingly, and unstable mortality regression estimates have been removed to avoid overinterpretation of findings derived from a very small number of events.

**7-** On validation. The heading “Additional validation of multivariable logistic regression models” is misleading, as no validation appears to have been performed. If the section is retained, the heading should be changed to something more accurate, such as “Apparent model discrimination”. However, this section may be unnecessary if the ROC and AUC analyses are removed.

**Response:** We thank the reviewer for this helpful suggestion. We agree that the previous section heading could be misleading, as no formal validation was performed. In the revised manuscript, the entire section describing ROC and AUC analyses has been removed, together with Figure 2. The Statistical Analysis, Results, and Discussion sections were revised accordingly to avoid predictive-model framing and to emphasize the exploratory nature of the multivariable analyses.

**8-** The Acknowledgements disclose the use of QuillBot Premium for language enhancement. This is acceptable in principle, but the statement remains broad. Please clarify the nature and extent of use, including which sections were edited, whether the tool was used only for grammar and style, and whether any generative AI tool was used at any stage of drafting, analysis, interpretation, or response preparation.

**Response:** We thank the reviewer for this important comment. We have revised the Acknowledgements section to provide a clearer and more detailed description of the use of QuillBot Premium during manuscript preparation. Specifically, we clarified that the tool was used exclusively for grammar correction, language refinement, and stylistic editing, and that it was not used for the generation of scientific content, data analysis, interpretation of results, or preparation of reviewer responses. No generative AI tool was used for data analysis, interpretation of results, generation of scientific content, or preparation of reviewer responses. The revised statement now reads as follows:

,,We would like to acknowledge the use of QuillBot Premium for language enhancement and editing assistance during manuscript preparation. This use complies with PLOS ONE's policy on AI-assisted writing tools. The tool was employed solely for grammar correction, linguistic refinement, and stylistic improvement of selected sections of the manuscript, and did not contribute to the generation, analysis, or interpretation of scientific content, nor to the preparation of reviewer responses. The authors take full responsibility for the content of the manuscript.”

**9-** The Conclusions should be tightened. The strongest conclusions relate to the concentration of severe and fatal disease among very young unvaccinated infants, the absence of maternal pertussis vaccination in the cohort, and the importance of preventive strategies targeting early infancy. Predictive-model claims should not feature in the Conclusions unless the models are validated.

**Response:** We thank the reviewer for this important comment. In response, we revised the Conclusions section to better align with the descriptive and exploratory nature of the study findings. Predictive-model framing and references to risk stratification were removed. The revised Conclusions now emphasize the concentration of severe and fatal pertussis among very young unvaccinated infants, the absence of maternal pertussis vaccination in the cohort, and the importance of preventive strategies targeting early infancy, particularly maternal vaccination.

**10-** Please check the manuscript for any remaining wording that implies clinical applicability of the models, and ensure that all figure references, table footnotes, and section headings are accurate.

**Response:** We thank the reviewer for this careful review of the manuscript. We have performed an additional comprehensive revision of the text to remove remaining wording implying clinical applicability or predictive utility of the models. We also carefully reviewed and corrected figure references, table footnotes, and section headings throughout the manuscript to ensure consistency and accuracy.
