# Supplementary material for: Clinical features, disease severity, and outcomes of pertussis in hospitalized children: A retrospective study from Serbia
Source: PLoS One. 2026 Jun 26;21(6):e0352498. doi: 10.1371/journal.pone.0352498 (PMC13308863; doi:10.1371/journal.pone.0352498)
Supplement: Peer review history

# Peer review: Decision Letter 0

**DOI:** 10.1371/journal.pone.0352498.r001

**Authors:** Daniela Hozbor

16 Mar 2026

PONE-D-25-67780

Pertussis outbreak in Serbia – is it finally time to change the national vaccination policy?

PLOS One

Dear Dr. Sovtic

Thank you for submitting your manuscript to PLOS ONE. After careful consideration, we feel that it has merit but does not fully meet PLOS ONE’s publication criteria as it currently stands. Therefore, we invite you to submit a revised version of the manuscript that addresses all the points raised during the review process.

Please submit your revised manuscript by Apr 30 2026 11:59PM. If you will need more time than this to complete your revisions, please reply to this message or contact the journal office at plosone@plos.org. When you're ready to submit your revision, log on to https://www.editorialmanager.com/pone/ and select the 'Submissions Needing Revision' folder to locate your manuscript file.

Please include the following items when submitting your revised manuscript:

- A letter that responds to each point raised by the academic editor and reviewer(s). You should upload this letter as a separate file labeled 'Response to Reviewers'.
- A marked-up copy of your manuscript that highlights changes made to the original version. You should upload this as a separate file labeled 'Revised Manuscript with Track Changes'.
- An unmarked version of your revised paper without tracked changes. You should upload this as a separate file labeled 'Manuscript'.

If you would like to make changes to your financial disclosure, please include your updated statement in your cover letter. Guidelines for resubmitting your figure files are available below the reviewer comments at the end of this letter.

If applicable, we recommend that you deposit your laboratory protocols in protocols.io to enhance the reproducibility of your results. Protocols.io assigns your protocol its own identifier (DOI) so that it can be cited independently in the future. For instructions see: https://journals.plos.org/plosone/s/submission-guidelines#loc-laboratory-protocols. Additionally, PLOS ONE offers an option for publishing peer-reviewed Lab Protocol articles, which describe protocols hosted on protocols.io. Read more information on sharing protocols at https://plos.org/protocols?utm_medium=editorial-email&utm_source=authorletters&utm_campaign=protocols.

We look forward to receiving your revised manuscript.

Kind regards,

Daniela Flavia Hozbor

Academic Editor

PLOS One

Journal Requirements:

When submitting your revision, we need you to address these additional requirements.

1. Please ensure that your manuscript meets PLOS ONE's style requirements, including those for file naming. The PLOS ONE style templates can be found at

https://journals.plos.org/plosone/s/file?id=wjVg/PLOSOne_formatting_sample_main_body.pdf and

https://journals.plos.org/plosone/s/file?id=ba62/PLOSOne_formatting_sample_title_authors_affiliations.pdf

2. We note that your Data Availability Statement is currently as follows: [All relevant data are within the manuscript and its Supporting Information files.]

Please confirm at this time whether or not your submission contains all raw data required to replicate the results of your study. Authors must share the “minimal data set” for their submission. PLOS defines the minimal data set to consist of the data required to replicate all study findings reported in the article, as well as related metadata and methods (https://journals.plos.org/plosone/s/data-availability#loc-minimal-data-set-definition).

For example, authors should submit the following data:

- The values behind the means, standard deviations and other measures reported;

- The values used to build graphs;

- The points extracted from images for analysis.

Authors do not need to submit their entire data set if only a portion of the data was used in the reported study.

If your submission does not contain these data, please either upload them as Supporting Information files or deposit them to a stable, public repository and provide us with the relevant URLs, DOIs, or accession numbers. For a list of recommended repositories, please see https://journals.plos.org/plosone/s/recommended-repositories.

If there are ethical or legal restrictions on sharing a de-identified data set, please explain them in detail (e.g., data contain potentially sensitive information, data are owned by a third-party organization, etc.) and who has imposed them (e.g., an ethics committee). Please also provide contact information for a data access committee, ethics committee, or other institutional body to which data requests may be sent. If data are owned by a third party, please indicate how others may request data access.

3. When completing the data availability statement of the submission form, you indicated that you will make your data available on acceptance. We strongly recommend all authors decide on a data sharing plan before acceptance, as the process can be lengthy and hold up publication timelines. Please note that, though access restrictions are acceptable now, your entire data will need to be made freely accessible if your manuscript is accepted for publication. This policy applies to all data except where public deposition would breach compliance with the protocol approved by your research ethics board. If you are unable to adhere to our open data policy, please kindly revise your statement to explain your reasoning and we will seek the editor's input on an exemption. Please be assured that, once you have provided your new statement, the assessment of your exemption will not hold up the peer review process.

4. If the reviewer comments include a recommendation to cite specific previously published works, please review and evaluate these publications to determine whether they are relevant and should be cited. There is no requirement to cite these works unless the editor has indicated otherwise.

[Note: HTML markup is below. Please do not edit.]

Reviewers' comments:

Reviewer's Responses to Questions

**Comments to the Author**

1. Is the manuscript technically sound, and do the data support the conclusions?

The manuscript must describe a technically sound piece of scientific research with data that supports the conclusions. Experiments must have been conducted rigorously, with appropriate controls, replication, and sample sizes. The conclusions must be drawn appropriately based on the data presented.

Reviewer #1: Yes

Reviewer #2: No

**********

2. Has the statistical analysis been performed appropriately and rigorously?

Reviewer #1: Yes

Reviewer #2: No

**********

3. Have the authors made all data underlying the findings in their manuscript fully available?

The PLOS Data policy requires authors to make all data underlying the findings described in their manuscript fully available without restriction, with rare exception (please refer to the Data Availability Statement in the manuscript PDF file). The data should be provided as part of the manuscript or its supporting information, or deposited to a public repository. For example, in addition to summary statistics, the data points behind means, medians and variance measures should be available. If there are restrictions on publicly sharing data—e.g. participant privacy or use of data from a third party—those must be specified.

Reviewer #1: Yes

Reviewer #2: No

**********

4. Is the manuscript presented in an intelligible fashion and written in standard English?

PLOS ONE does not copyedit accepted manuscripts, so the language in submitted articles must be clear, correct, and unambiguous. Any typographical or grammatical errors should be corrected at revision, so please note any specific errors here.

Reviewer #1: Yes

Reviewer #2: No

**********

5. Review Comments to the Author

Please use the space provided to explain your answers to the questions above. You may also include additional comments for the author, including concerns about dual publication, research ethics, or publication ethics. (Please upload your review as an attachment if it exceeds 20,000 characters)

Reviewer #1: Thanks for inviting me to review this retrospective single-center study of pertussis infections in children treated at a national referral center in Serbia between 2015 and 2025. While the manuscript overall is mostly well written and the data support the conclusions there are some specific areas of ambiguity/weakness that need to be addressed by the authors:

1) The title is a little misleading and should be revised- this is not a report of an outbreak of pertussis or a vaccine policy review. The title should be revised, a more appropriate title could be 'Predictors of mortality and disease severity amongst children with pertussis in Serbia'

2) In the Introduction (line 87-89) the authors state 'the pertussis booster vaccination for pregnant women is recommended... although it is not included in the mandatory schedule'. This is confusing. Do the authors mean that pertussis vaccination for pregnant women is recommended but that uptake is low? Please clarify, and if data are available about vaccine uptake for pregnant women (and the general population) in Serbia, it would be important to include.

3) The introduction should be strengthened to include references and additional context regarding recent concerning increases in pertussis cases and outbreaks in the US/Europe and as note by the WHO and also concerning declines in vaccination rates in some countries (see this article for a relevant summary- Vargas-Zambrano JC, Abrudan S, Macina D. Understanding the Epidemiology and Contributing Factors of Post-COVID-19 Pertussis Outbreaks: A Narrative Review. Infect Dis Ther. 2026 Jan;15(1):19-41. doi: 10.1007/s40121-025-01277-1. Epub 2025 Dec 1. PMID: 41324875; PMCID: PMC12783469.)

4) Methods- line 113-115- please clarify whether the patients included were consecutive patients with pertussis, all patients between the dates mentioned, or were selected in another way (e.g. at random)? How were eligible patients with pertussis identified (e.g. via PCR/serology results, or via a case registry, or via discharge diagnosis data?)?

5) Methods- please describe how positive serology was defined (especially for those who had received vaccination previously) and what serologic test was used.

6) Methods- please given a definition for fully/partially vaccinated in the methods, prior to discussing the results for this metric in line 210. Please also give a clear definition for prematurity.

7) Results- the first paragraph is

8) Results- Table 1 includes vaccination status or the infant/child. Was data regarding maternal vaccination status (i.e. pertussis booster during pregnancy) available? If yes, please include this. If no, then please add this to the discussion as a major limitation of the study

9) Results- line 214- it is not clear what is statistically different here and what the authors mean by 'favoring term births'. Do the authors mean that severe pertussis cases or deaths were significantly frequent among pre-term infants? Please clarify.

10) Results- line 217-218- 'The proportion of unvaccinated patients was significantly higher'- I am not sure what the comparator here is. Please clarify. Do you mean that among severe case of pertussis the proportion unvaccinated was significantly higher?

11) Results- line 228- I am not clear what the p-value of 0.016 refers to. Please clarify. Was apnea significantly more likely than the vomiting or cyanosis, or was it that a significant majority of cases had at least one additional symptom?

12) Discussion- if data about maternal pertussis vaccine booster during pregnancy was not available, please add this to the section discussing Study Limitations.

13) Conclusion- the authors state that 'pertussis outbreaks can still occur', but actually data from WHO and others (see the paper referenced above by Vargas-Zambrano et al) note worrying trends of increased outbreaks/cases in recent years. I recommend re-framing the beginning of the conclusion to remind the reader of the increased public health concern regarding rising pertussis outbreaks/cases in recent years across multiple countries.

More minor suggestions for improvement include the following:

1) Suggest using MV as abbreviation for mechanical ventilation instead of IMV to improve readability.

2) The methods section devotes a lot of space to describing the protocol for routine clinic care for pertussis at the center conducting the study (lines 118-183). This could be abbreviated/summarized.

3) Consider removing the p-values from Table 1- I am not sure they are very meaningful, except for the duration of cough differences which are already explored in the main results section text.

4) Table 2- please state whether the numbers in brackets are the range or interquartile range for each parameter.

5) Results- Line 213- there is a typo and 'first two 10 weeks' should read 'first 10 weeks' I think.

6) Please capitalize the 'f' in figure throughout where you are referring to a specific figure in the manuscript e.g. in line 320.

Reviewer #2: The topic is timely, the setting is clinically meaningful, and the manuscript has the potential to make a useful contribution. The manuscript contains some important descriptive findings, especially the observation that all fatal cases occurred in unvaccinated infants younger than 2 months, and that severe outcomes clustered heavily in very young infants and preterm children. These are meaningful observations and clinically worth reporting.

That said, the manuscript in its current form needs careful revision. My main concerns relate to internal consistency of the reported data, interpretation of the regression analyses, overstatement of model performance, and a title/conclusion that currently go further than the data can comfortably support.

1- At present, there are some obvious inconsistencies that need correction before the findings can be interpreted with confidence. The most striking example is in Table 1, where the vomiting row is not plausible as presented: the overall number is given as 18/107, but the subgroup values are listed as 66/80, 15/16, and 8/11. This cannot be correct and suggests either row displacement or transcription error. Similarly, Table 2 contains values that appear internally impossible, for example a leukocyte median of 27.34 with a range of 8.22–19.55. The same concern applies to other laboratory values in that table. These are not minor formatting issues, because they directly affect trust in the dataset and in the subsequent analyses. Please re-check all tables, denominators, ranges, and corresponding narrative statements very carefully.

2- I understand the intention to identify early predictors of deterioration, and this is clinically worthwhile. However, the number of severe outcomes is very small: 13 PICU admissions, 12 cases requiring invasive mechanical ventilation, and 5 deaths. With events this limited, the models become very unstable, especially when multiple candidate predictors are entered. This instability is visible in Table 3, where some odds ratios are extremely large and have very wide confidence intervals. For mortality in particular, the estimates for N/L ratio and coinfection are extraordinarily imprecise, which suggests sparse-data bias and overfitting rather than robust effect estimation. The use of Firth’s method is sensible, but it does not fully solve the underlying limitation of very few outcome events. I would strongly encourage the authors to simplify the models, reduce the number of predictors, and frame these analyses much more cautiously as exploratory rather than definitive.

3- Some interpretations of the regression findings do not match the reported effect estimates. This needs close checking throughout. For example, in the pneumonia model, the manuscript states that a higher N/L ratio predicted higher odds of pneumonia, but the reported odds ratio is 0.66. An odds ratio below 1 indicates lower odds, not higher odds. This is important because readers will follow the narrative interpretation, and at the moment there is a mismatch between the statistical output and the wording used to explain it. I would recommend a line-by-line check of every regression statement in the Results and Discussion sections against the actual coefficients and direction of association in the table.

4- The claims regarding model performance are too strong for a small retrospective single-centre cohort. The manuscript presents the PICU and IMV models as having excellent or outstanding discrimination, based on AUC values of 0.951 and 0.987, and goes on to describe this as confirmation of model robustness. I think that language is too strong. These performance estimates appear to come from the same dataset used to build the models, and there is no internal validation reported. In a small dataset with very few events, apparent AUC values are often optimistic. Without bootstrapping, cross-validation, or external validation, these models should not be described as robust predictive tools. The results are interesting and hypothesis-generating, but they need a much more restrained interpretation.

5- The title and policy framing should be moderated. The manuscript is based on a retrospective cohort of hospitalised children from a single tertiary centre. That design can certainly provide useful clinical and epidemiological insight, but it does not directly evaluate national vaccination policy, programme effectiveness, maternal vaccine uptake, or population-level prevention strategies. The current title asks whether it is time to change national vaccination policy, and the conclusion states that redefining national immunisation strategies may be the most effective means of protecting vulnerable age groups. I understand why the authors are making this connection, especially given that all fatal cases occurred in infants too young to be vaccinated. However, the study itself provides supportive observational evidence rather than direct policy evaluation. I think the paper would be stronger if the policy implications were framed more cautiously, as one reasonable interpretation of the observed vulnerability in early infancy rather than as a conclusion that follows directly from the data.

6- The study population needs clearer description, particularly in relation to age and vaccination status. The manuscript repeatedly frames the cohort as “infants and young children”, and the median age overall is 2 months. However, Table 1 shows that the fully vaccinated group had a median age of 6 years. This means that the vaccination groups are clearly not age-comparable, which is expected in pertussis, but it makes group comparisons difficult to interpret. For example, differences in cough duration and symptom profile across vaccination groups may simply reflect age structure rather than vaccine effect. This needs to be acknowledged more explicitly, and the manuscript should avoid overinterpreting group differences where age is such a strong confounder.

7. The ethics and consent description needs clarification. The manuscript states that this was a retrospective review of records from 2015 to 2025, approved in November 2025, and that written informed consent for the use of medical data was obtained from parents or legal guardians in accordance with institutional policy. This may be entirely appropriate locally, but as written it raises questions for the reader because retrospective chart reviews often rely on waiver or institutional approval for secondary use rather than fresh written consent. Please clarify how consent operated in practice here. Was consent already part of institutional admission procedures, or was there a specific ethics process for retrospective use of the data? This should be made fully transparent.

**********

6. PLOS authors have the option to publish the peer review history of their article (what does this mean?). If published, this will include your full peer review and any attached files.

If you choose “no”, your identity will remain anonymous but your review may still be made public.

**Do you want your identity to be public for this peer review?**  For information about this choice, including consent withdrawal, please see our Privacy Policy.

Reviewer #1: No

Reviewer #2: **Yes:** Dr Mamdooh Alzyood

**********

[NOTE: If reviewer comments were submitted as an attachment file, they will be attached to this email and accessible via the submission site. Please log into your account, locate the manuscript record, and check for the action link "View Attachments". If this link does not appear, there are no attachment files.]

To ensure your figures meet our technical requirements, please review our figure guidelines: https://journals.plos.org/plosone/s/figures

You may also use PLOS’s free figure tool, NAAS, to help you prepare publication quality figures: https://journals.plos.org/plosone/s/figures#loc-tools-for-figure-preparation.

NAAS will assess whether your figures meet our technical requirements by comparing each figure against our figure specifications.

# Author comment: Author response to Decision Letter 1

**DOI:** 10.1371/journal.pone.0352498.r002

2 Apr 2026

We thank the Editor and reviewers for their constructive and insightful comments. We have carefully revised the manuscript accordingly, including clarification of methodological aspects, re-evaluation of statistical analyses, and improvements in consistency between the Results, Discussion, and Abstract.

In addition, in accordance with PLOS ONE data availability requirements, we have provided a de-identified dataset as Supporting Information (S1 Dataset).

Detailed point-by-point responses to all reviewer comments are provided in a separate document.

Attachment Submitted filename: Response to Reviewers.docx

# Peer review: Decision Letter 1

**DOI:** 10.1371/journal.pone.0352498.r003

**Authors:** Daniela Hozbor, Daniela Hozbor

26 Apr 2026

PONE-D-25-67780R1

Clinical features, disease severity, and outcomes of pertussis in hospitalized children: a retrospective study from Serbia

PLOS One

Dear Dr. Sovtic,

Thank you for submitting your manuscript to PLOS ONE. After careful consideration, we feel that it has merit but does not fully meet PLOS ONE’s publication criteria as it currently stands. Therefore, we invite you to submit a revised version of the manuscript that addresses the points raised during the review process (reviewer#2).

Please submit your revised manuscript by Jun 10 2026 11:59PM. If you will need more time than this to complete your revisions, please reply to this message or contact the journal office at plosone@plos.org. When you're ready to submit your revision, log on to https://www.editorialmanager.com/pone/ and select the 'Submissions Needing Revision' folder to locate your manuscript file.

Please include the following items when submitting your revised manuscript:

- A letter that responds to each point raised by the academic editor and reviewer(s). You should upload this letter as a separate file labeled 'Response to Reviewers'.
- A marked-up copy of your manuscript that highlights changes made to the original version. You should upload this as a separate file labeled 'Revised Manuscript with Track Changes'.
- An unmarked version of your revised paper without tracked changes. You should upload this as a separate file labeled 'Manuscript'.

If you would like to make changes to your financial disclosure, please include your updated statement in your cover letter. Guidelines for resubmitting your figure files are available below the reviewer comments at the end of this letter.

If applicable, we recommend that you deposit your laboratory protocols in protocols.io to enhance the reproducibility of your results. Protocols.io assigns your protocol its own identifier (DOI) so that it can be cited independently in the future. For instructions see: https://journals.plos.org/plosone/s/submission-guidelines#loc-laboratory-protocols. Additionally, PLOS ONE offers an option for publishing peer-reviewed Lab Protocol articles, which describe protocols hosted on protocols.io. Read more information on sharing protocols at https://plos.org/protocols?utm_medium=editorial-email&utm_source=authorletters&utm_campaign=protocols.

As the corresponding author, your ORCID iD is verified in the submission system and will appear in the published article. PLOS supports the use of ORCID, and we encourage all coauthors to register for an ORCID iD and use it as well. Please encourage your coauthors to verify their ORCID iD within the submission system before final acceptance, as unverified ORCID iDs will not appear in the published article. *Only*  the individual author can complete the verification step; PLOS staff *cannot*  verify ORCID iDs on behalf of authors.

We look forward to receiving your revised manuscript.

Kind regards,

Daniela Flavia Hozbor

Academic Editor

PLOS One

Journal Requirements:

1. If the reviewer comments include a recommendation to cite specific previously published works, please review and evaluate these publications to determine whether they are relevant and should be cited. There is no requirement to cite these works unless the editor has indicated otherwise.

2. Please review your reference list to ensure that it is complete and correct. If you have cited papers that have been retracted, please include the rationale for doing so in the manuscript text, or remove these references and replace them with relevant current references. Any changes to the reference list should be mentioned in the rebuttal letter that accompanies your revised manuscript. If you need to cite a retracted article, indicate the article’s retracted status in the References list and also include a citation and full reference for the retraction notice.

[Note: HTML markup is below. Please do not edit.]

Reviewers' comments:

Reviewer's Responses to Questions

**Comments to the Author**

1. If the authors have adequately addressed your comments raised in a previous round of review and you feel that this manuscript is now acceptable for publication, you may indicate that here to bypass the “Comments to the Author” section, enter your conflict of interest statement in the “Confidential to Editor” section, and submit your "Accept" recommendation.

Reviewer #1: All comments have been addressed

Reviewer #2: All comments have been addressed

**********

2. Is the manuscript technically sound, and do the data support the conclusions?

The manuscript must describe a technically sound piece of scientific research with data that supports the conclusions. Experiments must have been conducted rigorously, with appropriate controls, replication, and sample sizes. The conclusions must be drawn appropriately based on the data presented.

Reviewer #1: Yes

Reviewer #2: Yes

**********

3. Has the statistical analysis been performed appropriately and rigorously?

Reviewer #1: Yes

Reviewer #2: I Don't Know

**********

4. Have the authors made all data underlying the findings in their manuscript fully available?

The PLOS Data policy requires authors to make all data underlying the findings described in their manuscript fully available without restriction, with rare exception (please refer to the Data Availability Statement in the manuscript PDF file). The data should be provided as part of the manuscript or its supporting information, or deposited to a public repository. For example, in addition to summary statistics, the data points behind means, medians and variance measures should be available. If there are restrictions on publicly sharing data—e.g. participant privacy or use of data from a third party—those must be specified.

Reviewer #1: Yes

Reviewer #2: Yes

**********

5. Is the manuscript presented in an intelligible fashion and written in standard English?

PLOS ONE does not copyedit accepted manuscripts, so the language in submitted articles must be clear, correct, and unambiguous. Any typographical or grammatical errors should be corrected at revision, so please note any specific errors here.

Reviewer #1: Yes

Reviewer #2: Yes

**********

6. Review Comments to the Author

Please use the space provided to explain your answers to the questions above. You may also include additional comments for the author, including concerns about dual publication, research ethics, or publication ethics. (Please upload your review as an attachment if it exceeds 20,000 characters)

Reviewer #1: The authors have addressed all of my previous concerns/questions. The results section in particular is much more clearly written in this revised manuscript and definitions for seropositivity and vaccination status of mothers has been added which also improves clarity.

Reviewer #2: The authors have responded constructively to the previous round of review and the manuscript is substantially improved. The core findings are clinically meaningful and the analytical concerns raised by Reviewer 2 have been addressed in good faith. The following comments should be addressed before acceptance.

1- The Discussion still contains clinical utility language suggesting the models "may assist clinicians in identifying patients" and "guiding early management decisions." Given that no internal or external validation has been performed, this should be softened. Add a clear statement that prospective validation is required before any clinical application can be considered.

2- The odds ratio for viral coinfection and mortality (47.1, 95% CI 2.5–888.1) is reported in Table 3 with a dash in place of a p-value, and no explanation for its absence. Either provide the p-value or add a brief note clarifying why it is not reported (for example, that Firth's penalized regression was used and conventional p-values are not applicable in this context).

3- The CI width for the coinfection-mortality association (2.5 to 888.1) means the data provide almost no information about the true effect size. Describing this finding as "exploratory" is not sufficient. The authors should state plainly that this estimate is statistically uninformative and should not be interpreted as evidence of a strong effect.

4- Table 1: confirm that the corrected vomiting row values (18/107 overall, 14/80, 1/16, 3/11) are accurate and consistent with the dataset. The subgroup values now sum correctly, but this should be verified against the source data given prior transcription errors in this table.

5- Table 2: the corrected leukocyte IQR within 72 hours (19.51–40.56) is now plausible against the reported median of 27.34. However, it would be worth adding a footnote clarifying that follow-up measurements were not available for all patients, if that is the case, to avoid readers assuming complete data.

6- The sentence in the Results reporting the PICU model fit (Nagelkerke R² = 0.472) and the MV model (Nagelkerke R² = 0.518) should be accompanied by a brief acknowledgement that pseudo-R² values in logistic regression are not directly comparable to R² in linear models and should be interpreted with caution, particularly in small samples.

7- The authors note that vaccination status was excluded from multivariable models due to collinearity with age, which is the correct decision. However, the Results section still includes a comparison of paroxysmal cough prevalence across vaccination groups (85% unvaccinated vs 54.5% fully vaccinated, p=0.039) and interprets this as a vaccine effect. Given the median age of 6 years in the fully vaccinated group versus 1.75 months in the unvaccinated group, this difference almost certainly reflects age rather than vaccination. The interpretation should be revised accordingly.

8- The Acknowledgements disclose the use of QuillBot Premium for language editing. Authors should confirm that this use is consistent with PLOS ONE's policy on AI-assisted writing tools, and if required, provide clarification on the extent of its use.

9- Minor: "Figure" should be capitalised consistently throughout the manuscript when referring to a specific figure. A few instances remain where it appears in lowercase.

Thank you.

**********

7. PLOS authors have the option to publish the peer review history of their article (what does this mean?). If published, this will include your full peer review and any attached files.

If you choose “no”, your identity will remain anonymous but your review may still be made public.

**Do you want your identity to be public for this peer review?**  For information about this choice, including consent withdrawal, please see our Privacy Policy.

Reviewer #1: No

Reviewer #2: **Yes:** Dr Mamdooh Alzyood

**********

[NOTE: If reviewer comments were submitted as an attachment file, they will be attached to this email and accessible via the submission site. Please log into your account, locate the manuscript record, and check for the action link "View Attachments". If this link does not appear, there are no attachment files.]

To ensure your figures meet our technical requirements, please review our figure guidelines: https://journals.plos.org/plosone/s/figures

You may also use PLOS’s free figure tool, NAAS, to help you prepare publication quality figures: https://journals.plos.org/plosone/s/figures#loc-tools-for-figure-preparation.

NAAS will assess whether your figures meet our technical requirements by comparing each figure against our figure specifications.

# Author comment: Author response to Decision Letter 2

**DOI:** 10.1371/journal.pone.0352498.r004

5 May 2026

We thank the reviewers for the careful evaluation of our manuscript and for the constructive comments. We have revised the manuscript accordingly and addressed all points raised. All changes are reflected in the revised version of the manuscript.

Reviewer #2: The authors have responded constructively to the previous round of review and the manuscript is substantially improved. The core findings are clinically meaningful and the analytical concerns raised by Reviewer 2 have been addressed in good faith. The following comments should be addressed before acceptance.

1- The Discussion still contains clinical utility language suggesting the models "may assist clinicians in identifying patients" and "guiding early management decisions." Given that no internal or external validation has been performed, this should be softened. Add a clear statement that prospective validation is required before any clinical application can be considered.

Response: Thank you for this comment. We agree that the clinical utility of the predictive models should be further tempered given the lack of internal and external validation. We have revised the Discussion to remove language implying clinical application and added a clear statement that prospective validation in independent cohorts is required before any clinical use can be considered. The relevant sentences have been rephrased accordingly.

2- The odds ratio for viral coinfection and mortality (47.1, 95% CI 2.5–888.1) is reported in Table 3 with a dash in place of a p-value, and no explanation for its absence. Either provide the p-value or add a brief note clarifying why it is not reported (for example, that Firth's penalized regression was used and conventional p-values are not applicable in this context).

Response: We thank the reviewer for this comment. Given the use of penalized regression, we focused on reporting effect estimates and confidence intervals, which are considered more informative in this setting. We have clarified this in the revised manuscript by adding an explanatory footnote to Table 3 and adjusting the table formatting accordingly.

3- The CI width for the coinfection-mortality association (2.5 to 888.1) means the data provide almost no information about the true effect size. Describing this finding as "exploratory" is not sufficient. The authors should state plainly that this estimate is statistically uninformative and should not be interpreted as evidence of a strong effect.

Response: We thank the reviewer for this important comment. We agree that the very wide confidence interval reflects substantial imprecision due to the small number of events. In the revised manuscript, we have explicitly clarified that the estimated odds ratio should not be interpreted as evidence of a strong effect and emphasized the limited robustness of these findings. The text has been revised accordingly.

4- Table 1: confirm that the corrected vomiting row values (18/107 overall, 14/80, 1/16, 3/11) are accurate and consistent with the dataset. The subgroup values now sum correctly, but this should be verified against the source data given prior transcription errors in this table.

Response: We thank the reviewer for this comment. We confirm that the corrected vomiting row values are accurate and consistent with the dataset. The previous issue resulted from a coding error in the initial table version, which has now been corrected and verified against the source data.

5- Table 2: the corrected leukocyte IQR within 72 hours (19.51–40.56) is now plausible against the reported median of 27.34. However, it would be worth adding a footnote clarifying that follow-up measurements were not available for all patients, if that is the case, to avoid readers assuming complete data.

Response: Thank you for this helpful comment. We have clarified that follow-up laboratory measurements within 72 hours were not available for all patients. Specifically, we have added a statement in the Results section indicating the number of patients with available follow-up data (76/107), and included a corresponding footnote in Table 2 to indicate incomplete data availability.

6- The sentence in the Results reporting the PICU model fit (Nagelkerke R² = 0.472) and the MV model (Nagelkerke R² = 0.518) should be accompanied by a brief acknowledgement that pseudo-R² values in logistic regression are not directly comparable to R² in linear models and should be interpreted with caution, particularly in small samples.

Response: We thank the reviewer for this important comment. In response, we have revised the Results section to include a statement clarifying that pseudo-R² measures in logistic regression are not directly comparable to R² from linear regression models and should be interpreted with caution, especially in the context of small sample sizes.

7- The authors note that vaccination status was excluded from multivariable models due to collinearity with age, which is the correct decision. However, the Results section still includes a comparison of paroxysmal cough prevalence across vaccination groups (85% unvaccinated vs 54.5% fully vaccinated, p=0.039) and interprets this as a vaccine effect. Given the median age of 6 years in the fully vaccinated group versus 1.75 months in the unvaccinated group, this difference almost certainly reflects age rather than vaccination. The interpretation should be revised accordingly.

Response: Thank you for this important comment. We agree that the observed difference in paroxysmal cough prevalence across vaccination groups is confounded by substantial differences in age distribution between groups. We have revised the Results section to remove any causal interpretation and to clarify that this finding most likely reflects age differences rather than an independent effect of vaccination.

8- The Acknowledgements disclose the use of QuillBot Premium for language editing. Authors should confirm that this use is consistent with PLOS ONE's policy on AI-assisted writing tools, and if required, provide clarification on the extent of its use.

Response: We thank the reviewer for this observation. We confirm that the use of QuillBot Premium complies with the policy of PLOS ONE regarding AI-assisted writing tools. The tool was used solely for linguistic refinement and did not contribute to the generation or interpretation of scientific content. The Acknowledgements section has been revised accordingly. The authors retain full responsibility for the integrity and accuracy of the work.

9- Minor: "Figure" should be capitalised consistently throughout the manuscript when referring to a specific figure. A few instances remain where it appears in lowercase.

Response: We thank the reviewer for this comment. We have carefully reviewed the manuscript to ensure consistent capitalization of the term “Figure” when referring to specific figures, and have confirmed that it is used consistently throughout the text.

Attachment Submitted filename: Response to the reviewers - round 2.docx

# Peer review: Decision Letter 2

**DOI:** 10.1371/journal.pone.0352498.r005

**Authors:** Daniela Hozbor, Daniela Hozbor, Daniela Hozbor

24 May 2026

PONE-D-25-67780R2

Clinical features, disease severity, and outcomes of pertussis in hospitalized children: a retrospective study from Serbia

PLOS One

Dear Dr. Aleksandar Sovtic,

Thank you for submitting your manuscript to PLOS ONE. After careful consideration, we feel that it has merit but does not fully meet PLOS ONE’s publication criteria as it currently stands. Therefore, we invite you to submit a revised version of the manuscript that addresses the points raised by reviewer#2  during the review process.

Please submit your revised manuscript by Jul 08 2026 11:59PM. If you will need more time than this to complete your revisions, please reply to this message or contact the journal office at plosone@plos.org. When you're ready to submit your revision, log on to https://www.editorialmanager.com/pone/ and select the 'Submissions Needing Revision' folder to locate your manuscript file.

Please include the following items when submitting your revised manuscript:

- A letter that responds to each point raised by the academic editor and reviewer(s). You should upload this letter as a separate file labeled 'Response to Reviewers'.
- A marked-up copy of your manuscript that highlights changes made to the original version. You should upload this as a separate file labeled 'Revised Manuscript with Track Changes'.
- An unmarked version of your revised paper without tracked changes. You should upload this as a separate file labeled 'Manuscript'.

If you would like to make changes to your financial disclosure, please include your updated statement in your cover letter. Guidelines for resubmitting your figure files are available below the reviewer comments at the end of this letter.

If applicable, we recommend that you deposit your laboratory protocols in protocols.io to enhance the reproducibility of your results. Protocols.io assigns your protocol its own identifier (DOI) so that it can be cited independently in the future. For instructions see: https://journals.plos.org/plosone/s/submission-guidelines#loc-laboratory-protocols. Additionally, PLOS ONE offers an option for publishing peer-reviewed Lab Protocol articles, which describe protocols hosted on protocols.io. Read more information on sharing protocols at https://plos.org/protocols?utm_medium=editorial-email&utm_source=authorletters&utm_campaign=protocols.

As the corresponding author, your ORCID iD is verified in the submission system and will appear in the published article. PLOS supports the use of ORCID, and we encourage all coauthors to register for an ORCID iD and use it as well. Please encourage your coauthors to verify their ORCID iD within the submission system before final acceptance, as unverified ORCID iDs will not appear in the published article. *Only*  the individual author can complete the verification step; PLOS staff *cannot*  verify ORCID iDs on behalf of authors.

We look forward to receiving your revised manuscript.

Kind regards,

Daniela Flavia Hozbor

Academic Editor

PLOS One

Journal Requirements:

If the reviewer comments include a recommendation to cite specific previously published works, please review and evaluate these publications to determine whether they are relevant and should be cited. There is no requirement to cite these works unless the editor has indicated otherwise.

Please review your reference list to ensure that it is complete and correct. If you have cited papers that have been retracted, please include the rationale for doing so in the manuscript text, or remove these references and replace them with relevant current references. Any changes to the reference list should be mentioned in the rebuttal letter that accompanies your revised manuscript. If you need to cite a retracted article, indicate the article’s retracted status in the References list and also include a citation and full reference for the retraction notice.

[Note: HTML markup is below. Please do not edit.]

Reviewers' comments:

Reviewer's Responses to Questions

**Comments to the Author**

1. If the authors have adequately addressed your comments raised in a previous round of review and you feel that this manuscript is now acceptable for publication, you may indicate that here to bypass the “Comments to the Author” section, enter your conflict of interest statement in the “Confidential to Editor” section, and submit your "Accept" recommendation.

Reviewer #2: All comments have been addressed

**********

2. Is the manuscript technically sound, and do the data support the conclusions?

The manuscript must describe a technically sound piece of scientific research with data that supports the conclusions. Experiments must have been conducted rigorously, with appropriate controls, replication, and sample sizes. The conclusions must be drawn appropriately based on the data presented.

Reviewer #2: Partly

**********

3. Has the statistical analysis been performed appropriately and rigorously?

Reviewer #2: I Don't Know

**********

4. Have the authors made all data underlying the findings in their manuscript fully available?

The PLOS Data policy requires authors to make all data underlying the findings described in their manuscript fully available without restriction, with rare exception (please refer to the Data Availability Statement in the manuscript PDF file). The data should be provided as part of the manuscript or its supporting information, or deposited to a public repository. For example, in addition to summary statistics, the data points behind means, medians and variance measures should be available. If there are restrictions on publicly sharing data—e.g. participant privacy or use of data from a third party—those must be specified.

Reviewer #2: Yes

**********

5. Is the manuscript presented in an intelligible fashion and written in standard English?

PLOS ONE does not copyedit accepted manuscripts, so the language in submitted articles must be clear, correct, and unambiguous. Any typographical or grammatical errors should be corrected at revision, so please note any specific errors here.

Reviewer #2: Yes

**********

6. Review Comments to the Author

Please use the space provided to explain your answers to the questions above. You may also include additional comments for the author, including concerns about dual publication, research ethics, or publication ethics. (Please upload your review as an attachment if it exceeds 20,000 characters)

Reviewer #2: The authors have responded constructively to the previous review, and the manuscript has improved. The clinical dataset is valuable, particularly as a single-centre retrospective account of severe pertussis in hospitalised children in Serbia. However, several issues still require revision before the manuscript is ready for publication.

1- On mortality analysis. The mortality analysis remains the main concern. There were only five deaths, all occurring in unvaccinated infants younger than two months, and all with documented viral coinfection. The reported odds ratio for viral coinfection and mortality, 47.1 with a 95% CI of 2.5 to 888.1, is too imprecise to be clinically or statistically informative. I recommend removing this estimate from Table 3 and presenting the mortality findings descriptively in the Results. The descriptive pattern is itself important and does not require regression modelling.

2- On AUC and ROC analysis. The reported AUC values for PICU admission and mechanical ventilation are apparent AUCs calculated from the same dataset used to develop the models. No internal or external validation has been performed. These values may therefore overestimate model performance. I recommend either performing internal validation, for example using bootstrap optimism correction, or removing the AUC values and ROC figure. Given the sample size and event numbers, removing them and reframing the models as exploratory association analyses would be the more appropriate option.

3- On prediction-model framing. The manuscript still gives the impression that the models have predictive or clinical risk-stratification value. This should be softened throughout. The models should be described as exploratory and hypothesis-generating only. Any wording implying clinical use, early identification for practice, or management guidance should be removed unless the models are properly validated.

4- On number of outcome events. The models for PICU admission and mechanical ventilation are based on small numbers of events: 13 PICU admissions and 12 cases requiring mechanical ventilation. This creates a risk of overfitting and unstable estimates. This limitation should be stated more clearly in the Results and Discussion.

5- On prematurity estimates. The associations between prematurity and severe outcomes are clinically plausible, but the confidence intervals are very wide. For example, the estimates for PICU admission and mechanical ventilation span more than an order of magnitude. The authors should avoid presenting the point estimates as precise. Phrases such as “more than a 20-fold increase in risk” should be qualified or revised.

6- Table 3 should be revised. I recommend removing the mortality column or limiting it to descriptive information. If the authors retain the table, it should not present unstable mortality estimates in a way that suggests reliable inference.

7- On validation. The heading “Additional validation of multivariable logistic regression models” is misleading, as no validation appears to have been performed. If the section is retained, the heading should be changed to something more accurate, such as “Apparent model discrimination”. However, this section may be unnecessary if the ROC and AUC analyses are removed.

8- The Acknowledgements disclose the use of QuillBot Premium for language enhancement. This is acceptable in principle, but the statement remains broad. Please clarify the nature and extent of use, including which sections were edited, whether the tool was used only for grammar and style, and whether any generative AI tool was used at any stage of drafting, analysis, interpretation, or response preparation.

9- The Conclusions should be tightened. The strongest conclusions relate to the concentration of severe and fatal disease among very young unvaccinated infants, the absence of maternal pertussis vaccination in the cohort, and the importance of preventive strategies targeting early infancy. Predictive-model claims should not feature in the Conclusions unless the models are validated.

10- Please check the manuscript for any remaining wording that implies clinical applicability of the models, and ensure that all figure references, table footnotes, and section headings are accurate.

**********

7. PLOS authors have the option to publish the peer review history of their article (what does this mean?). If published, this will include your full peer review and any attached files.

If you choose “no”, your identity will remain anonymous but your review may still be made public.

**Do you want your identity to be public for this peer review?**  For information about this choice, including consent withdrawal, please see our Privacy Policy.

Reviewer #2: **Yes:** Dr Mamdooh Alzyood

**********

[NOTE: If reviewer comments were submitted as an attachment file, they will be attached to this email and accessible via the submission site. Please log into your account, locate the manuscript record, and check for the action link "View Attachments". If this link does not appear, there are no attachment files.]

To ensure your figures meet our technical requirements, please review our figure guidelines: https://journals.plos.org/plosone/s/figures

You may also use PLOS’s free figure tool, NAAS, to help you prepare publication quality figures: https://journals.plos.org/plosone/s/figures#loc-tools-for-figure-preparation.

NAAS will assess whether your figures meet our technical requirements by comparing each figure against our figure specifications.

# Author comment: Author response to Decision Letter 3

**DOI:** 10.1371/journal.pone.0352498.r006

28 May 2026

Reviewer #2: The authors have responded constructively to the previous review, and the manuscript has improved. The clinical dataset is valuable, particularly as a single-centre retrospective account of severe pertussis in hospitalised children in Serbia. However, several issues still require revision before the manuscript is ready for publication.

1- On mortality analysis. The mortality analysis remains the main concern. There were only five deaths, all occurring in unvaccinated infants younger than two months, and all with documented viral coinfection. The reported odds ratio for viral coinfection and mortality, 47.1 with a 95% CI of 2.5 to 888.1, is too imprecise to be clinically or statistically informative. I recommend removing this estimate from Table 3 and presenting the mortality findings descriptively in the Results. The descriptive pattern is itself important and does not require regression modelling.

Response: We thank the reviewer for this important comment. We agree that the mortality analysis was limited by the very small number of fatal events, resulting in unstable and imprecise regression estimates. In response, we removed the mortality regression estimates from Table 3 and revised the corresponding Results section to present mortality findings descriptively rather than through regression modelling. Specifically, references to continuity-corrected odds ratios and penalized logistic regression were removed. The revised text now emphasizes the observed clinical pattern and exploratory findings without suggesting reliable effect estimation.

2- On AUC and ROC analysis. The reported AUC values for PICU admission and mechanical ventilation are apparent AUCs calculated from the same dataset used to develop the models. No internal or external validation has been performed. These values may therefore overestimate model performance. I recommend either performing internal validation, for example using bootstrap optimism correction, or removing the AUC values and ROC figure. Given the sample size and event numbers, removing them and reframing the models as exploratory association analyses would be the more appropriate option.

Response: We thank the reviewer for this important comment. We agree that the reported AUC values represented apparent performance estimates derived from the same dataset used for model development and therefore could overestimate true model performance. Given the limited sample size and number of outcome events, we decided not to pursue formal internal validation. Instead, in accordance with the reviewer’s recommendation, we removed the ROC analyses, AUC values, and Figure 2 from the manuscript and reframed the multivariable analyses as exploratory association analyses rather than predictive models.

3- On prediction-model framing. The manuscript still gives the impression that the models have predictive or clinical risk-stratification value. This should be softened throughout. The models should be described as exploratory and hypothesis-generating only. Any wording implying clinical use, early identification for practice, or management guidance should be removed unless the models are properly validated.

Response: We thank the reviewer for this important comment. In response, we revised the manuscript throughout to soften predictive framing and to emphasize the exploratory nature of the multivariable analyses. References implying clinical applicability, risk stratification, or management guidance were removed or rephrased. The models are now consistently described as exploratory and hypothesis-generating, and the corresponding sections in the Statistical Analysis, Results, Discussion, and Conclusions were revised accordingly.

4- On number of outcome events. The models for PICU admission and mechanical ventilation are based on small numbers of events: 13 PICU admissions and 12 cases requiring mechanical ventilation. This creates a risk of overfitting and unstable estimates. This limitation should be stated more clearly in the Results and Discussion.

Response: We thank the reviewer for this important observation. We agree that the relatively limited number of severe outcome events, particularly PICU admission and mechanical ventilation, increases the risk of overfitting and unstable estimates. In response, we added explicit statements in both the Results and Discussion sections emphasizing that the multivariable analyses should be interpreted cautiously and considered exploratory and hypothesis-generating.

5- On prematurity estimates. The associations between prematurity and severe outcomes are clinically plausible, but the confidence intervals are very wide. For example, the estimates for PICU admission and mechanical ventilation span more than an order of magnitude. The authors should avoid presenting the point estimates as precise. Phrases such as “more than a 20-fold increase in risk” should be qualified or revised.

Response: We thank the reviewer for this valuable comment. We agree that the confidence intervals surrounding the prematurity estimates were wide and that the point estimates should not be interpreted as precise measures of effect size. Accordingly, we revised the Results and Discussion sections to remove wording suggesting precise magnitude of risk and added clarifying statements emphasizing the imprecision and cautious interpretation of these estimates.

6- Table 3 should be revised. I recommend removing the mortality column or limiting it to descriptive information. If the authors retain the table, it should not present unstable mortality estimates in a way that suggests reliable inference.

Response: We thank the reviewer for this helpful suggestion. Table 3 has been revised accordingly, and unstable mortality regression estimates have been removed to avoid overinterpretation of findings derived from a very small number of events.

7- On validation. The heading “Additional validation of multivariable logistic regression models” is misleading, as no validation appears to have been performed. If the section is retained, the heading should be changed to something more accurate, such as “Apparent model discrimination”. However, this section may be unnecessary if the ROC and AUC analyses are removed.

Response: We thank the reviewer for this helpful suggestion. We agree that the previous section heading could be misleading, as no formal validation was performed. In the revised manuscript, the entire section describing ROC and AUC analyses has been removed, together with Figure 2. The Statistical Analysis, Results, and Discussion sections were revised accordingly to avoid predictive-model framing and to emphasize the exploratory nature of the multivariable analyses.

8- The Acknowledgements disclose the use of QuillBot Premium for language enhancement. This is acceptable in principle, but the statement remains broad. Please clarify the nature and extent of use, including which sections were edited, whether the tool was used only for grammar and style, and whether any generative AI tool was used at any stage of drafting, analysis, interpretation, or response preparation.

Response: We thank the reviewer for this important comment. We have revised the Acknowledgements section to provide a clearer and more detailed description of the use of QuillBot Premium during manuscript preparation. Specifically, we clarified that the tool was used exclusively for grammar correction, language refinement, and stylistic editing, and that it was not used for the generation of scientific content, data analysis, interpretation of results, or preparation of reviewer responses. No generative AI tool was used for data analysis, interpretation of results, generation of scientific content, or preparation of reviewer responses. The revised statement now reads as follows:

,,We would like to acknowledge the use of QuillBot Premium for language enhancement and editing assistance during manuscript preparation. This use complies with PLOS ONE's policy on AI-assisted writing tools. The tool was employed solely for grammar correction, linguistic refinement, and stylistic improvement of selected sections of the manuscript, and did not contribute to the generation, analysis, or interpretation of scientific content, nor to the preparation of reviewer responses. The authors take full responsibility for the content of the manuscript.”

9- The Conclusions should be tightened. The strongest conclusions relate to the concentration of severe and fatal disease among very young unvaccinated infants, the absence of maternal pertussis vaccination in the cohort, and the importance of preventive strategies targeting early infancy. Predictive-model claims should not feature in the Conclusions unless the models are validated.

Response: We thank the reviewer for this important comment. In response, we revised the Conclusions section to better align with the descriptive and exploratory nature of the study findings. Predictive-model framing and references to risk stratification were removed. The revised Conclusions now emphasize the concentration of severe and fatal pertussis among very young unvaccinated infants, the absence of maternal pertussis vaccination in the cohort, and the importance of preventive strategies targeting early infancy, particularly maternal vaccination.

10- Please check the manuscript for any remaining wording that implies clinical applicability of the models, and ensure that all figure references, table footnotes, and section headings are accurate.

Response: We thank the reviewer for this careful review of the manuscript. We have performed an additional comprehensive revision of the text to remove remaining wording implying clinical applicability or predictive utility of the models. We also carefully reviewed and corrected figure references, table footnotes, and section headings throughout the manuscript to ensure consistency and accuracy.

Attachment Submitted filename: Response to the reviewer.docx

# Peer review: Decision Letter 3

**DOI:** 10.1371/journal.pone.0352498.r007

**Authors:** Daniela Hozbor, Daniela Hozbor, Daniela Hozbor, Daniela Hozbor

11 Jun 2026

Clinical features, disease severity, and outcomes of pertussis in hospitalized children: a retrospective study from Serbia

PONE-D-25-67780R3

Dear Dr. Aleksandar Sovtic,

We’re pleased to inform you that your manuscript has been judged scientifically suitable for publication and will be formally accepted for publication once it meets all outstanding technical requirements.

Within one week, you’ll receive an e-mail detailing the required amendments. When these have been addressed, you’ll receive a formal acceptance letter and your manuscript will be scheduled for publication.

An invoice will be generated when your article is formally accepted. Please note, if your institution has a publishing partnership with PLOS and your article meets the relevant criteria, all or part of your publication costs will be covered. Please make sure your user information is up-to-date by logging into Editorial Manager at Editorial Manager® and clicking the ‘Update My Information' link at the top of the page. For questions related to billing, please contact billing support.

If your institution or institutions have a press office, please notify them about your upcoming paper to help maximize its impact. If they’ll be preparing press materials, please inform our press team as soon as possible -- no later than 48 hours after receiving the formal acceptance. Your manuscript will remain under strict press embargo until 2 pm Eastern Time on the date of publication. For more information, please contact onepress@plos.org.

Kind regards,

Daniela Flavia Hozbor

Academic Editor

PLOS One

Additional Editor Comments (optional):

Reviewers' comments:

Reviewer's Responses to Questions

**Comments to the Author**

1. If the authors have adequately addressed your comments raised in a previous round of review and you feel that this manuscript is now acceptable for publication, you may indicate that here to bypass the “Comments to the Author” section, enter your conflict of interest statement in the “Confidential to Editor” section, and submit your "Accept" recommendation.

Reviewer #2: All comments have been addressed

**********

2. Is the manuscript technically sound, and do the data support the conclusions?

The manuscript must describe a technically sound piece of scientific research with data that supports the conclusions. Experiments must have been conducted rigorously, with appropriate controls, replication, and sample sizes. The conclusions must be drawn appropriately based on the data presented.

Reviewer #2: Yes

**********

3. Has the statistical analysis been performed appropriately and rigorously?

Reviewer #2: I Don't Know

**********

4. Have the authors made all data underlying the findings in their manuscript fully available?

The PLOS Data policy requires authors to make all data underlying the findings described in their manuscript fully available without restriction, with rare exception (please refer to the Data Availability Statement in the manuscript PDF file). The data should be provided as part of the manuscript or its supporting information, or deposited to a public repository. For example, in addition to summary statistics, the data points behind means, medians and variance measures should be available. If there are restrictions on publicly sharing data—e.g. participant privacy or use of data from a third party—those must be specified.

Reviewer #2: Yes

**********

5. Is the manuscript presented in an intelligible fashion and written in standard English?

PLOS ONE does not copyedit accepted manuscripts, so the language in submitted articles must be clear, correct, and unambiguous. Any typographical or grammatical errors should be corrected at revision, so please note any specific errors here.

Reviewer #2: Yes

**********

6. Review Comments to the Author

Please use the space provided to explain your answers to the questions above. You may also include additional comments for the author, including concerns about dual publication, research ethics, or publication ethics. (Please upload your review as an attachment if it exceeds 20,000 characters)

Reviewer #2: The authors have responded constructively to the previous comments and the manuscript has improved substantially. The main methodological concerns have now been addressed, particularly the removal of unstable mortality regression estimates, the removal of ROC/AUC analyses, the clearer framing of the regression models as exploratory and hypothesis-generating, and the clarification of limitations related to the small number of severe outcomes. The revised manuscript now presents the key findings more appropriately, with the strongest conclusions focused on the concentration of severe and fatal pertussis among very young unvaccinated infants and the importance of preventive strategies, including maternal vaccination.

I have no further major concerns. I recommend acceptance. Before final publication, the authors may wish to ensure that any remaining wording implying clinical prediction or direct risk-stratification utility is softened, particularly in the Abstract and Conclusion, but this is an editorial issue rather than a barrier to publication.

**********

7. PLOS authors have the option to publish the peer review history of their article (what does this mean?). If published, this will include your full peer review and any attached files.

If you choose “no”, your identity will remain anonymous but your review may still be made public.

**Do you want your identity to be public for this peer review?**  For information about this choice, including consent withdrawal, please see our Privacy Policy.

Reviewer #2: **Yes:** Dr Mamdooh Alzyood

**********

# Editor report: Acceptance letter

**DOI:** 10.1371/journal.pone.0352498.r008

**Authors:** Daniela Hozbor, Daniela Hozbor, Daniela Hozbor, Daniela Hozbor

PONE-D-25-67780R3

PLOS One

Dear Dr. Sovtic,

I'm pleased to inform you that your manuscript has been deemed suitable for publication in PLOS One. Congratulations! Your manuscript is now being handed over to our production team.

At this stage, our production department will prepare your paper for publication. This includes ensuring the following:

* All references, tables, and figures are properly cited

* All relevant supporting information is included in the manuscript submission,

* There are no issues that prevent the paper from being properly typeset

You will receive further instructions from the production team, including instructions on how to review your proof when it is ready. Please keep in mind that we are working through a large volume of accepted articles, so please give us a few days to review your paper and let you know the next and final steps.

Lastly, if your institution or institutions have a press office, please let them know about your upcoming paper now to help maximize its impact. If they'll be preparing press materials, please inform our press team within the next 48 hours. Your manuscript will remain under strict press embargo until 2 pm Eastern Time on the date of publication. For more information, please contact onepress@plos.org.

You will receive an invoice from PLOS for your publication fee after your manuscript has reached the completed accept phase. If you receive an email requesting payment before acceptance or for any other service, this may be a phishing scheme. Learn how to identify phishing emails and protect your accounts at https://explore.plos.org/phishing.

If we can help with anything else, please email us at customercare@plos.org.

Thank you for submitting your work to PLOS ONE and supporting open access.

Kind regards,

PLOS ONE Editorial Office Staff

on behalf of

Dr. Daniela Flavia Hozbor

Academic Editor

PLOS One
